# Nitrogen dioxide reductions from satellite and surface observations during COVID-19 mitigation in Rome (Italy)

**DOI:** 10.1007/s11356-020-12141-9

**Published:** 2021-01-12

**Authors:** Cristiana Bassani, Francesca Vichi, Giulio Esposito, Mauro Montagnoli, Marco Giusto, Antonietta Ianniello

**Affiliations:** CNR - Institute of Atmospheric Pollution Research, Via Salaria Km 29.3, CP10, 00015 Monterotondo S., Rome, Italy

**Keywords:** COVID-19, Rome, Air pollution, Nitrogen dioxide, TROPOMI, Reduction

## Abstract

Lockdown restrictions were implemented in Italy from 10 March 2020 to contain the COVID-19 pandemic. Our study aims to evaluate air pollution changes, with focus on nitrogen dioxide (NO_2_), before and during the lockdown in Rome and in the surroundings. Significant NO_2_ declines were observed during the COVID-19 pandemic with reductions of − 50%, − 34%, and − 20% at urban traffic, urban background, and rural background stations, respectively. Tropospheric NO_2_ vertical column density (VCD) from the TROPOspheric Monitoring Instrument (TROPOMI) was used to evaluate the spatial-temporal variations of the NO_2_ before and during the lockdown for the entire area where the surface stations are located. The evaluation is concerned with the pixels including one or more air quality stations to explore the capability of the unprecedented high spatial resolution to monitor urban and rural sites from space with relation to the surface measurements. Good agreement between surface concentration and TROPOMI VCD was obtained in Rome (*R* = 0.64 in 2019, *R* = 0.77 in 2020) and in rural sites (*R* = 0.71 in 2019). Inversely, a slight correlation (*R* = 0.20) was observed in rural areas during the lockdown due to very low levels of NO_2_. Finally, the TROPOMI VCD showed a sharp decline in NO_2_, larger in urban (− 43%) than in rural sites (− 17%) as retrieved with the concurrent surface measurements averaging all the traffic and urban background (− 44%) and all the rural background stations (− 20%). These results suggest air pollution improvement in Rome gained from implementing lockdown restrictions.

**Supplementary Information:**

The online version contains supplementary material available at 10.1007/s11356-020-12141-9.

## Introduction

Nitrogen oxides (NO_x_ = nitric oxide (NO) + nitrogen dioxide (NO_2_)) play a key role in the troposphere by producing ozone (O_3_) and secondary aerosol, and, thus, impacting human health, ecosystems, and climate change. NO_x_ are released in the troposphere mainly by anthropogenic sources. According to the European Environmental Agency (2019), the primary sources of nitrogen oxides in Europe are road transport sector, accounting for 39% of the emissions, followed by energy production and distribution sectors (16%), commercial, household, and institutional activities (14%), and energy use in industry (12%). The rest of the nitrogen oxide emissions in Europe comes from other sectors of minor relevance such as non-road transport (8%), agriculture (8%), and industrial processes and product use (3%). Thus, the largest NO_x_ source results in the fossil fuel combustion processes, which almost exclusively leads to emission directly into the atmosphere, mainly in the form of NO. NO is readily oxidized to the more hazardous NO_2_ (R1), which is photo-dissociated to NO and ground state atomic oxygen (O(^3^P)) (R2), which combines with molecular oxygen (O_2_) to give O_3_ (Seinfeld and Pandis 2006) (R3) through a termolecular reaction with a third body (M, e.g., N_2_). Once formed, O_3_ reacts with NO to regenerate NO_2_ in the absence of significant atmospheric ozone sources (carbon monoxide (CO) and volatile organic compounds (VOCs)) (Sillman 1995) and, hence, in the absence of competing interconversion reactions:

R1 $$ {\mathrm{O}}_3+\mathrm{NO}\to {\mathrm{NO}}_2+{\mathrm{O}}_2 $$

R2 $$ {\mathrm{NO}}_2+\mathrm{h}\upnu\ \left(<420\ \mathrm{nm}\right)\to \mathrm{NO}+\mathrm{O}\left({}^3\mathrm{P}\right) $$

R3 $$ \mathrm{O}\left({}^3\mathrm{P}\right)+{\mathrm{O}}_2+\mathrm{M}\to {\mathrm{O}}_3+\mathrm{M} $$

Thus, during the day, NO and NO_2_ concentrations are related to the O_3_ concentration in a photo-stationary state (PSS) null cycle. As a result, this rapid interconversion between the NO and NO_2_ allows them to be treated as NO_x_.

VOCs and CO, especially in urban areas or in polluted regions, alter this photo-stationary state cycle by changing the path of ozone formation through reactions with hydroxyl radical (OH) and forming intermediary compounds like hydroperoxyl (HO_2_) and organic peroxy (RO_2_) radicals, which in turn also oxide NO to NO_2_ (R4 and R5), without consuming ozone and providing additional NO_2_ (to R1). Hence, the subsequent NO_2_ photolysis (R2) followed by R3 results in a net source of ozone:

R4 $$ {\mathrm{HO}}_2+\mathrm{NO}\to \mathrm{OH}+{\mathrm{NO}}_2 $$

R5 $$ {\mathrm{RO}}_2+\mathrm{NO}\to \mathrm{RO}+{\mathrm{NO}}_2 $$

The priority of these reactions and, thus, the formation and retaining of ozone depend on the intensity of solar radiation, stratospheric-tropospheric exchange, long-range transport, and the ratios of different precursor species (NO_x_, CO, and VOCs) (Seinfeld and Pandis 2006; Jin and Holloway 2015; Sharma et al. 2017). It is well recognized that ozone is an important greenhouse gas and plays a key role in the photochemistry and oxidizing capacity of the troposphere, in addition to its detrimental effects on human health, crops, and vegetation (Seinfeld and Pandis 2006). However, the relation between O_3_, NO_x_, and VOCs is connected to a complex and highly non-linear photochemistry. Accordingly, there are two sensitivity regimes of O_3_ production, namely, the NO_x_-limited and VOC-limited regimes. In the NO_x_-limited regime, generally in rural areas (Jin et al. 2020), an increase in NO_x_ values leads to an increase in ozone production which shows to be only slightly affected by VOC variations. In the VOC-limited regime, generally in urban areas (Witte et al. 2011), ozone increases with increasing VOC and decreases with increasing NO_x_. Therefore, reductions in VOCs will only be effective in reducing ozone if VOC-limited chemistry predominates and reductions in NO_x_ will be effective in O_3_ reduction only if NO_x_-limited chemistry predominates.

Additionally, nitrogen dioxide is a toxic gas that can cause both long-term and short-term effects on health (Jonson et al. 2017; Kowalska et al. 2020; Manisalidis et al. 2020). Thus, the European Union (EU) has established the air quality guidelines and standards for the protection of human health. Limit values for NO_2_ are set at 200 μg/m^3^ for 1 h average concentrations, not to be exceeded more than 18 h/year, and 40 μg/m^3^ annual average concentrations (Directive 2008/50/EC). These limit values are in line with the World Health Organization (WHO) air quality guidelines (WHO 2006). In addition to this, the EU recommends the vegetation critical value of 30 μg/m^3^ annual average for NO_x_. Current trends across European air quality networks show that the annual limit value of NO_2_ is violated at many stations in sixteen of the EU Member States and four other countries (EEA 2019). The highest concentration above the annual NO_2_ limit value was observed not only at traffic stations but also at urban and rural background stations.

Due to contagion of Coronavirus disease (COVID-19), which is an infection disease initially identified in Wuhan, China, in December 2019 (Hui et al. 2020), leading to thousands of deaths and a rapid increase in cases worldwide, European countries, such as other regions of the world, have implemented lockdown restrictions on transportation, agricultural, and industrial activities to contain and stop the spread of the pandemic. Similarly, Italy imposed a lockdown on 8 March 2020 (DPCM 2020a) for northern regions and, then, on 10 March 2020 (DPCM 2020b) for the whole country, including restricted social distancing measures and reducing the mobility (traffic on the roads, air transport) and non-essential business and industrial activity countrywide. For this reason, in the period 23 February–27 March 2020, a 47% decrease in vehicular traffic in the Lazio region, up to 61% in the metropolitan area of Rome, was reported (Arpa Lazio 2020). In addition, the energy consumptions in the area comprising Lazio, Campania, and Abruzzo regions during the lockdown months (March and April 2020) dropped off by 7% and 17%, respectively (https://www.terna.it/it/sistema-elettrico/transparency-report/total-load (last access July 08, 2020)).

Despite the severe impacts of lockdown on people’s social life and global economy, there is increased interest in the improvement of air quality in Italy and across the world, being principally determined by emissions of atmospheric pollutants from human activities. Recent researches on surface measurements reported NO_2_ improvements associated to social distancing and traffic restrictions during the COVID-19 pandemic observing 20–61% reductions over China (Xu et al. 2020a), 18%–51% over India (Sharma et al. 2020; Jain and Sharma 2020), 25–30% over the USA (Berman and Ebisu 2020), and 20–30% over France and Italy (Muhammad et al. 2020).

As said before, NO_2_ is mainly emitted from burning fossil fuels (e.g., diesel, gasoline, coal) and, therefore, changes in NO_2_ concentrations can be used as an indicator of changes in levels of human activities (traffic, factories). In situations of prolonged lockdown and over time, the expectation is that average levels of NO_2_ will decrease. Therefore, understanding these temporary reductions in emissions provides a unique opportunity to evaluate how this has affected NO_2_ concentrations and how the atmosphere could respond to a possible future of alternative energy sources that will not emit this pollutant.

In this work, we examine the NO_2_ concentrations in Rome and in the northern surrounding rural areas before and during the Italian lockdown, to evaluate the air quality when a low NO_2_ level never reached before in the atmosphere over an urban site is expected. The evaluation of the air quality by surface data is completed considering NO_2_ and also NO, O_3_, and CO collected from 1 March to 30 April during 2019 and 2020 in all types of monitoring stations of Rome and surrounding areas to compare diurnally, monthly, and yearly changes and effects on air pollution before and during COVID-19 lockdown. The comparison is referred to different types of fixed stations, since traffic sites, urban and suburban backgrounds, and rural sites were considered. This allowed a wider comprehension of the air quality changes in relation to the drastic emissions reductions, and also provided a comparison with the satellite data collected during the same periods in various environments. Unfortunately, VOCs data were not available to be able to quantify the magnitude of the influence of these compounds on ozone variation levels, especially in the rural environment. However, considering that NO_x_ is a O_3_ precursor, it is important to evaluate the lockdown effects also on this pollutant examining its trends in both urban and rural areas.

The description of the spatial-temporal variation of the NO_2_ concentration includes the tropospheric NO_2_ columnar content provided by the TROPOMI (TROPOspheric Monitoring Instrument) sensor on board of Sentinel 5 Precursor (S5P) satellite (Veefkind et al. 2012). In the framework of Copernicus program, the S5P was launched in October 2017 by European Space Agency (ESA) to monitor the density of several compounds (e.g., NO_2_, CO, O_3_, CH_4_, CH_2_O) and cloud distributions affecting air quality and climate with higher spatial resolution (ground pixel at nadir 7 × 3.5 km^2^ before August 6, 2019, and then 5.5 × 3.5 km^2^, as reported in Verhoelst et al. 2020) than the other remote sensing sensors such as Ozone Monitoring Instrument (OMI) with a spatial resolution of 13 × 24 km^2^ (Krotkov et al. 2016; Levelt et al. 2006). Previous studies considered TROPOMI for its high spatial resolution to monitor air pollutants at urban level (Ialongo et al. 2020) and in remote areas with different types of industrial sources (Griffin et al. 2019). Recently, some papers (Tobías et al. 2020; Nakada and Urban 2020) reported a reduction in air pollution levels during the lockdown measures in Barcelona and Sao Paulo cities, respectively. Tobías et al. (2020) highlight a significant decrease of NO_2_ surface concentration and columnar content during the Spanish lockdown. The authors specify that the lockdown could be a factor affecting the reduction in conjunction with others such as meteorology and regional and long transport of pollutants. As reported in http://www.esa.int/Applications/Observing_the_Earth/Copernicus/Sentinel-5P/Coronavirus_lockdown_leading_to_drop_in_pollution_across_Europe (last accessed October 23, 2020), a reduction of the TROPOMI NO_2_ columnar content was observed with respect to the monthly average of March 2019 in the major European cities. Thus, we consider the tropospheric NO_2_ vertical density (VCD) available in the period from 10 March till 3 May 2019 and 2020 to observe the mitigation of the NO_2_ from space in Rome and the surrounding areas before and during the Italian lockdown. The analysis is performed by extracting the tropospheric NO_2_ VCD from the pixels where the air quality stations are located.

In the present work, atmospheric NO_2_ observations based on surface measurements and tropospheric TROPOMI VCD are shown and discussed in Rome and, for the first time, in the surroundings, taking also the opportunity of the unprecedented NO_2_ decrease as a result of lockdown restrictions to contain the COVID-19.

## Material and methods

### Site description

The area investigated includes the city of Rome and its surroundings and is extended to the north and north-eastern edge of the Lazio region characterized by the lower concentration of NO_2_ close to the polluted urban site as described in the annual reports of the Lazio Regional Agency for Environmental Protection (ARPA Lazio) in http://www.arpalazio.net/main/aria/doc/pubblicazioni.php.

Rome is the capital of Italy and the third most populous city in the European Union (about four million people), including Rome’s population in addition to adjacent suburban areas. International financial, cultural, and business center, Rome is characterized by several services, high-technology companies, research, construction and commercial activities (especially banking), and the huge development of tourism, which are important to its economy. However, the increase of activities, population, and urbanization together with Mediterranean climate, urban traffic, not sustainable urban mobility, and household heating led to several pollution episodes and health problems.

The regional area under exam moving from Rome downtown toward its outskirts (represented by Cavaliere and Guido monitoring stations), and further outside the metropolitan area, can be roughly divided into two main zones: coastal and Apenninic. In the coastal area, several transportation facilities are located, such as Fiumicino Airport and the harbors of Fiumicino and Civitavecchia. Fiumicino airport, located approximately 30 km south-west of Rome, is the first Italian international hub: in 2019, 43.5 million passengers and 194,500 tons of cargoes travelled through this infrastructure (https://assaeroporti.com, last accessed July 08, 2020). Maritime traffic in this area is mainly conveyed to the port of Civitavecchia which had a traffic of about 1.8 million passengers and 9.6 million tons of cargoes in 2019, whereas in the same period, about 3.5 million tons of cargoes passed through the port of Fiumicino, principally employed for oil products supply (https://www.portidiroma.it, last access July 08, 2020).

Following the Consular Roads, which depart from the center of Rome along north-west (Via Aurelia and Via Cassia) and north-east (Via Salaria) directions, several small towns within the region are connected to the capital. Via Aurelia is a coastal route along which Civitavecchia and, nearby, in a hilly area in proximity to the sea, Allumiere are located. Following Via Cassia the city of Viterbo and in the northernmost edge of this province, near the border to Tuscany, the small village of Acquapendente is situated.

Along the north-east direction, not far from the Via Salaria route, in the northern side of Mount Terminillo, part of the Apennines, the village of Leonessa is positioned at 969 m a.s.l., a few km from the border of Umbria. These areas are mainly devoted to tourism and agricultural activities. Allumiere, being nearer to the city of Rome and very close to Civitavecchia harbor, may be possibly more impacted by anthropogenic activities, but on the whole, the small villages mentioned can suitably represent the regional rural background. Therefore, Lazio Regional Agency for Environmental Protection (ARPA Lazio) has included the locations described in its network of fixed stations, which extensively monitors the air quality.

### Surface measurements

**Fig. 1:**
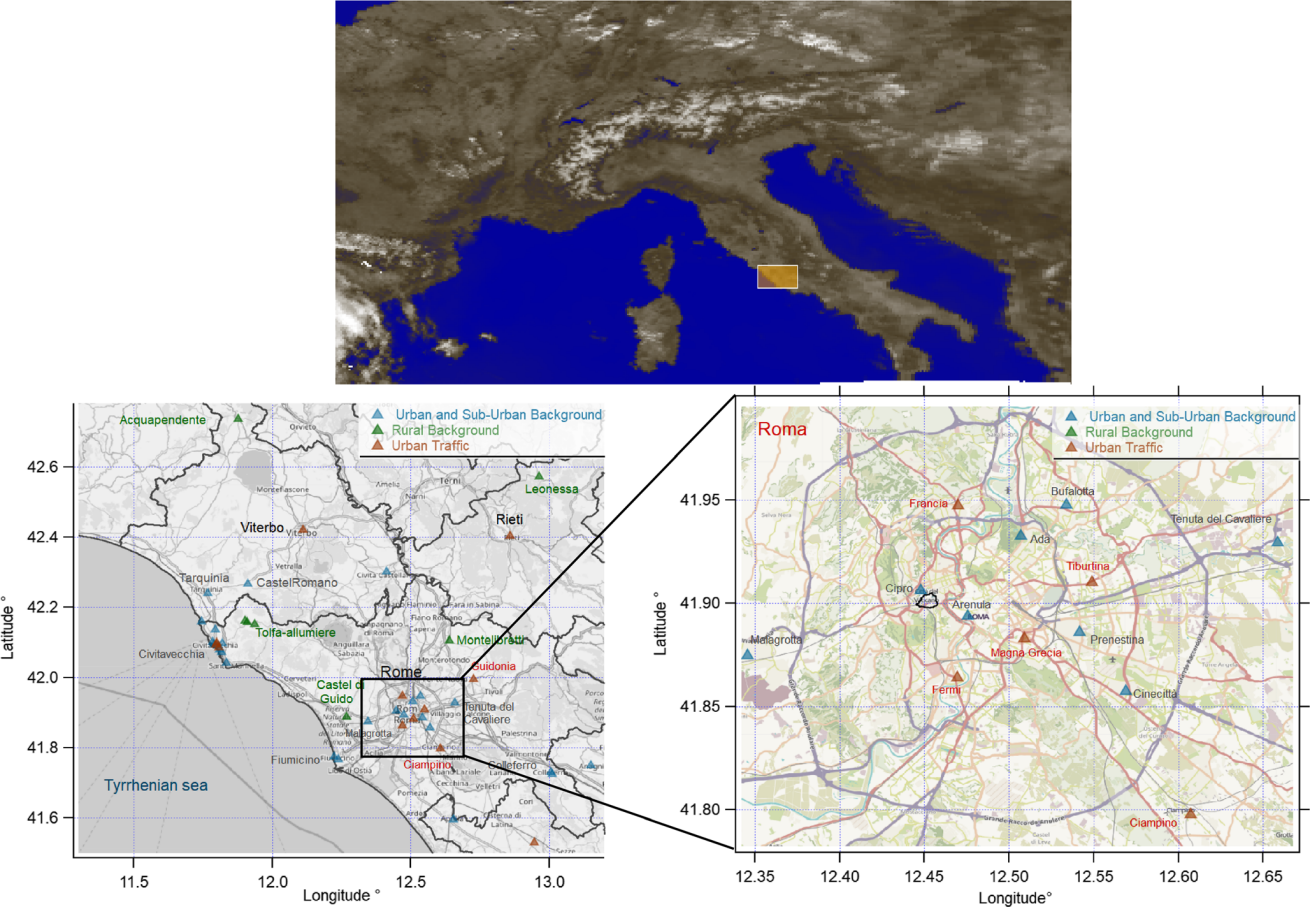
On the top, the central Italian region (yellow square) where the surface stations are enclosed (TROPOMI “scene albedo” product, 10/04/2020); in the bottom left, map of the location of monitoring stations in the area of Rome and in surrounding areas. On the bottom right, the zoom of Rome highlights the distribution of gardens and parks (green) in the city

Hourly NO_2_, NO, O_3_, and CO concentrations and weather conditions were provided from Lazio Regional Agency for Environmental Protection (ARPA Lazio), which makes an open data portal available to users (http://www.arpalazio.gov.it/ambiente/aria/), for six urban traffic (UT) (Francia, Magna Grecia, Fermi, Tiburtina, Ciampino, and Civ. Via Roma), seven urban background (UB) (Arenula, Preneste, Ada, Bufalotta, Cipro, Cinecittà, Fiumicino Guglielmi), two suburban background (SB) (Cavaliere, Malagrotta), and four rural background (RB) (Guido, Allumiere, Leonessa, Acquapendente) monitoring stations in Rome and regional territory. Figure 1 shows the area where all the mentioned stations are located (yellow subset) with the maps of the specific location of all the stations (bottom left) and specifically of the city of Rome (bottom right).

These monitoring stations were chosen as representative of NO_2_, NO, O_3_, and CO concentrations in Rome and its surrounding areas. The urban traffic (UT) stations are the ones for which the biggest variation is expected during the lockdown.

NO_2_ and NO measurements were carried out using a NO_x_ analyzer model 200A (Teledyne API Inc., detection limit of 0.53 μg/m^3^ and 0.82 μg/m^3^ for NO and NO_2_, respectively), based on chemiluminescence. Calibration of NO_x_ analyzer occurred at 04:00–05:00 daily and, hence, the measurements have been removed. O_3_ is monitored based on UV light absorption signal with a Teledyne API ozone analyzer model 400E (detection limit of 1.28 μg/m^3^). CO measurements were carried out using a CO gas filter correlation analyzer model 300E (Teledyne Advanced Pollution Instrumentation API Inc., detection limit < 0.05 mg/m^3^), based on nondispersive infrared spectroscopy.

The procedures and the control of the quality of the surface measurements (QA/QC) are assured by ARPA Lazio by internal protocols, which fully comply with the standards required by EC Directives: EN 14626:2012 (CEN 2012a) for CO, EN 14211:2012 (CEN 2012b) for NO and NO_2_, and EN 14625:2012 (CEN 2012c) for O_3_. These standards specify type-approval test procedures for demonstrating that the applied measurement method complies with the data quality objectives specified in Directive 2008/50/EC (ISPRA 2014; ARPA Lazio 2015). Furthermore, the work presented in this paper takes into account atmospheric measurements from 1 March to 30 April during 2019 and 2020 for each station to calculate the mean levels of each pollutant and, therefore, to evaluate and compare air pollution trends and variation levels prior to and during COVID-19 lockdown in Rome. A confidence interval of 95% is selected to calculate the mean of all the available data. Daily means for air pollutants have been calculated starting from hourly means measured at each site to assess the mean levels of each pollutant for each month and for each year. Thus, trend analysis was carried out after grouping the measurements carried out in each monitoring station into one averaged monitoring station pattern. Furthermore, the variations of mean concentration were calculated to assess relative difference (%) comparing the lockdown period (March–April 2020) to the same period of previous year 2019 for NO_2_, NO, CO, and O_3_. These variations are evaluated at each monitoring station applying the Welch two sample *t*-test to determine a 95% confidence (*p* < 0.05) statistical significance criteria for each pollutant and, thus, to discard or retain the obtained difference values for each pollutant monitored in that station during the lockdown period.

This study includes atmospheric measurements of NO_2_, NO, and O_3_ in A. Liberti station (42° 06′ 21″ N, 12° 38′ 25″ E) of the Institute of Atmospheric Pollution Research of National Research Council (CNR-IIA). This station is located at Montelibretti town, a rural area, located about 30 km north-east of Rome, and not directly affected by anthropogenic activities. This station is a research infrastructure employed for monitoring atmospheric processes which affect the dynamics of air pollutants. Same period of ARPA measurements for 2019 and 2020 was taken into account to determine NO_2_, NO, and O_3_ variations.

As already stated in the “Introduction” section, we focus our attention on NO_2_ concentrations considered proxies of pollution emissions due to human activities, first of all vehicular traffic.

### NO_2_ emission sources and their variation during the lockdown

For NO_2_, Table S1 reports the last available emission inventory of the area, referred to year 2015 (ARPA Lazio 2016). Data are disaggregated by macro-sectors and by territorial areas called “provinces.” Most of the monitoring stations considered in this study are located in the province of Rome, except for Acquapendente and Leonessa which are located in the provinces of Viterbo and Rieti, respectively.

It can be observed that, in the Rome area, road traffic contributes to more than 50% of total NO_2_ emissions. This sector has strongly decreased during the lockdown in March and April 2020. Such a reduction is well documented, for instance, by the average hourly car passes reported in Table S2: these car passes are constantly monitored at 6 different sites of the city by the Mobility Agency of the Rome Municipality (Roma Mobilità 2020). On average, a mean lockdown variation of − 67% was experienced, if data are compared to March and April 2019.

An even greater decrease has occurred for plane transportation in the Fiumicino airport, as shown in Table S3, with an average plane reduction of − 77% (Aeroporti di Roma 2020). Ship transportation has probably experienced a similar reduction, but data are only available on a half-yearly basis (MIT 2020). Consequently, Table S4 compares the period January–June 2020 with the same months of 2019: an overall reduction of ship transportation can be mainly observed for ferries and cruises (− 62% and − 83%, respectively) while minor variations are observed for freight transportation. However, it is important to remark that off-road transportation represents less than 14% of total NO_2_ emissions of the Rome province, as shown in the abovementioned Table S1. Consequently, the main variation in NO_2_ emissions is related to road traffic reduction.

Another major source of NO_2_ is the combustion in energy and industry plants. During the lockdown, electricity production in thermal plants has slightly decreased (− 19%): these data are only available on a National scale (Terna 2020) and are reported in Table S5. It can be assumed that a similar trend was also experienced by the energy plants located in the Rome area. No specific information is available on industrial plants activity during the lockdown. However, the electricity demand in the city of Rome decreased by 10.1% and 19.4% in March and April 2020, respectively, compared to the same months of 2019: this suggests that most of the industrial activities had only minor variations during the lockdown.

As a summary, the main variation in the emissions of NO_2_ during the lockdown is related to the reduction of road transportation.

### TROPOMI product

TROPOMI is a hyperspectral nadir-viewing sensor on board the Sentinel 5P satellite (S5P) (Veefkind et al. 2012) with a daily revisitation time (in Italy at ∼ 12:00 UTM). The TROPOMI instrument is composed of four spectrometers operating from ultraviolet (UV) to near-infrared (NIR) spectral domains. The NO_2_ columnar content (hereinafter, vertical column density, VCD) is provided for the total amount by the DOAS method (Platt and Stutz 2008) applied to the acquired upwelling radiance in the 405–465 nm spectral range and the solar irradiance (van Geffen et al. 2019). In addition, the tropospheric and stratospheric VCD are provided by the assimilation of the total VCD with the vertical distribution of the NO_2_ of the TM5-MP chemical transport model (van Geffen et al. 2019).

The NO_2_ products are available in near-real-time (NRTI) generated after a reprocessing of the data no later than 3 h and offline (OFFL) provided within 2 weeks from the acquisition time. The outcomes of the validation activities highlighted that these processing chains provide products with similar performance with a bias within the mission requirements (Lambert et al. 2019).

The tropospheric NO_2_ VCD is the TROPOMI product used to evaluate the NO_2_ reduction from space for the high variability of the pollutant occurring in the lower atmospheric layers, especially over urban sites taking advantage of the high spatial resolution (ground pixel at nadir 7 × 3.5 km^2^ before August 6, 2019, and then 5.5 × 3.5 km^2^, as reported in Verhoelst et al. 2020). The TROPOMI products are chosen in the central Italian region comprehensive of the urban and the surrounding rural areas (Fig. 1) where all the considered surface stations are located.

The OFFL tropospheric VCD are selected in lockdown period from 10 March to 3 May 2020 and also in the same period of 2019, downloaded from https://s5phub.copernicus.eu/dhus/#/home (last access: June 2, 2020). These products fulfill the status and the quality assurance (qa) of the product by screening the qa_value > 0.75 condition in all the ground pixels including the air quality stations, as recommended for all applications (van Geffen et al. 2019; Eskes et al. 2019).

The TROPOMI products are processed with SNAP (SeNtinel Application Platform), which is an open source architecture for the exploitation of Earth Observation data, software of the European Space Agency (http://step.esa.int/main/download/), developed by Brockmann Consult under the GPL (general public license) open source license. This platform is not designed specifically for remotely sensed atmospheric products but this kind of satellite data can be downloaded and processed on SNAP with small tricks to analyze the map of the atmospheric product and to extract data from TROPOMI netCDF (Network Common Data Form) file for a specific pixel.

## Results and discussion

### Meteorological conditions

Air pollutant levels can be affected by seasonal and/or local changes in the meteorological conditions, due to the expected differences between the city and the surrounding area meteorological data are discussed separately in the following section.

In urban areas, the meteorological data indicated that weather conditions during the lockdown period (March and April 2020) were almost similar to those of the same periods in the year 2019. During the months of March and April, the mean monthly global radiation in daylight hours, Rg, air temperature, T, relative humidity, RH, and wind speed were similar during both the years with little variations (Table 1 and Fig. S1-S2). The meteorological conditions during the period of interest in rural and suburban areas were also examined (Table 2 and Fig. S3-S6). As expected, the temperatures at rural sites were lower (about 0.7–0.8 °C) with respect to the city. The month of March 2019, if compared to March 2020, was characterized by higher pressure and higher temperatures. In particular, the days 23–24–25 March had both minimum and maximum temperatures lower in 2020 with respect to 2019. During the 25 March 2020, the minimum temperature ranged in the early morning from − 1.3 °C at Cavaliere to 1 °C in Guido, and the maximum ranged from 12 °C at Guido and Civitavecchia to 14 °C at Cavaliere and Montelibretti. The same was also observed for 1–2–3 April, particularly on 2 April when the minimum temperatures ranged from − 1.6 °C at Montelibretti to 1.3 °C at Guido, the maximum temperatures varied from 12 °C at Civitavecchia and Guido to 15 °C at Cavaliere and Montelibretti. The opposite happened in the period from 4 to 14 April when the average temperatures during 2020 raised to higher values if compared to 2019. Afterward, the 2 years exhibited comparable temperatures in the rest of the month of April. The main difference observed between the 2 years is in the rainfall regime, since precipitations were respectively less and more abundant during the months of March and April 2019, compared to the same months of 2020. The atmospheric pressure and average temperature were higher in April 2020 with respect to April 2019. The global radiation during March 2019 was higher than in March 2020, and, as expected, it increased in the month of April of both the years, being higher in 2020, if compared to 2019. A slight difference between the 2 months could be assessed: while March was characterized by good weather and conditions typical of higher pressure in 2019, April 2019 was more humid and rainy; hence, the global radiation did not have a strong impact.

**Table 1:** Monthly averages of temperature, relative humidity, wind speed, rainfall, pressure, and solar irradiance from March to April 2020 and from March to April 2019 in urban monitoring stations

Parameter	March 2019	March 2020	April 2019	April 2020
Temperature (°C)	13.2 ± 3.3	12.0 ± 3.4	14.7 ± 3.7	15.0 ± 4.0
Relative humidity (%)	65.8 ± 23.7	71.2 ± 22.1	70.9 ± 20.8	68.6 ± 25.1
Wind speed (m s^−1^)	1.8 ± 1.0	1.7 ± 1.1	1.7 ± 0.9	1.6 ± 0.8
Rainfall (mm)	2.7 ± 0.3	19.2 ± 0.3	32.6 ± 0.8	14.8 ± 0.4
Pressure (mbar)	1018.6 ± 4.2	1014.8 ± 7.1	1013.0 ± 5.9	1016.3 ± 5.1
Global radiation* (W m^−2^)	302.1 ± 250.1	303.6 ± 263.6	361.7 ± 268.2	406.7 ± 302.4

*Global radiation has been expressed as average global radiation in daylight hours

**Table 2:** Monthly averages of temperature, relative humidity, wind speed, rainfall, pressure, and solar irradiance from March to April 2019 and from March to April 2020 in suburban and rural monitoring stations

Parameter	March 2019	March 2020	April 2019	April 2020
Temperature (°C)	12.4 ± 3.7	11.4 ± 4.0	13.9 ± 4.0	14.5 ± 4.3
Relative humidity (%)	68.8 ± 21.2	68.7 ± 17.7	74.5 ± 17.6	68.8 ± 19.7
Wind speed (m s^−1^)	2.6 ± 3.2	2.5 ± 1.7	2.2 ± 3.0	2.1 ± 1.3
Rainfall (mm)	7.3 ± 1.5	23.4 ± 0.3	34.7 ± 2.1	15.2 ± 0.2
Pressure (mbar)	1017.1 ± 87.1	1013.0 ± 6.6	1011.5 ± 97.0	1014.8 ± 5.1
Global radiation* (W m^−2^)	298.7 ± 218.4	287.9 ± 219.7	360.9 ± 228.3	385.4 ± 255.3

*Global radiation has been expressed as average global radiation in daylight hours

**Fig. 2:**
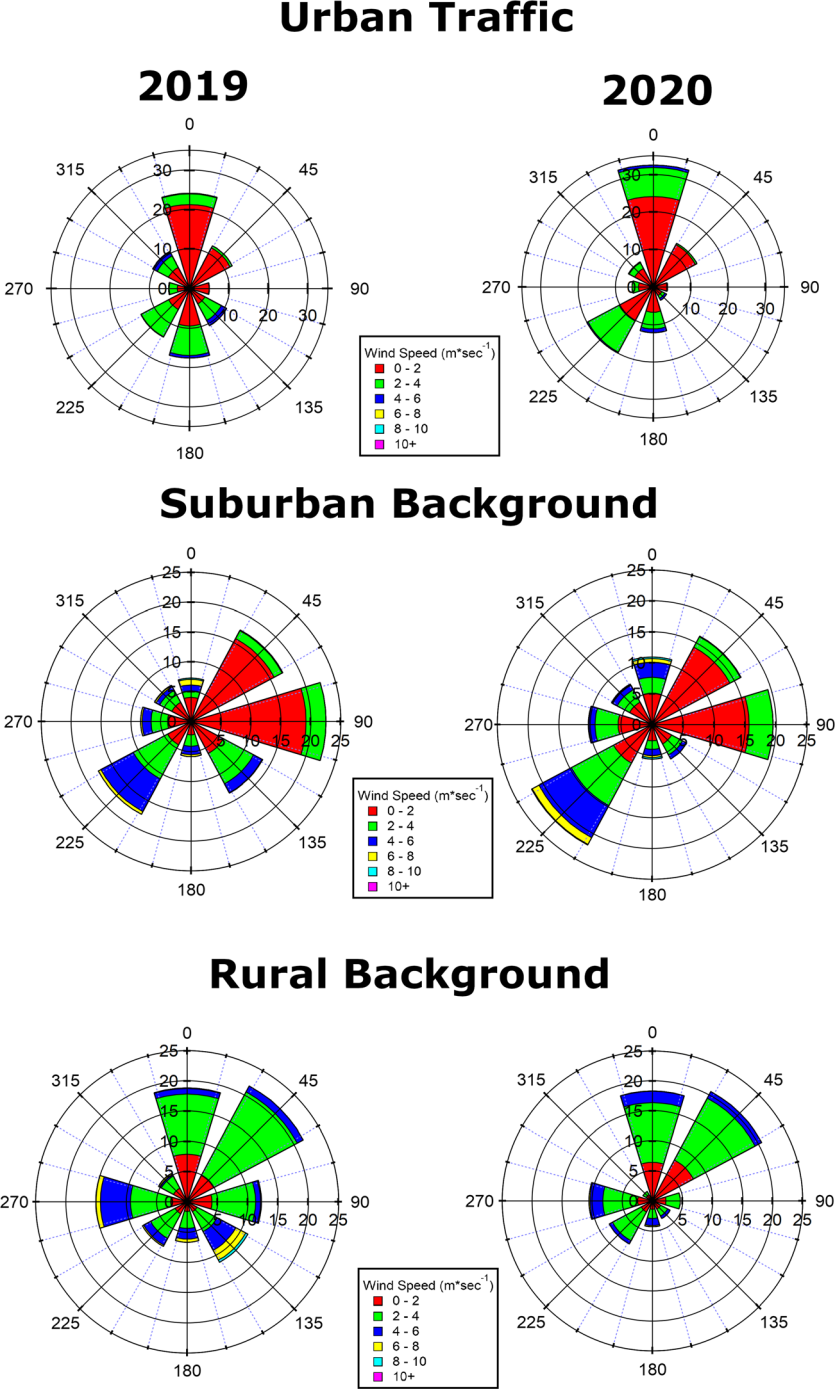
Wind roses showing the frequency distributions of wind directions and speeds (color scale) averaged over March–April 2019 and March–April 2020 in urban traffic and suburban and rural background monitoring stations in Rome. The radius axis represents the occurrence from 0 to 35% in urban traffic and from 0 to 25% in suburban and rural stations

Taking into account the wind records at three different types of monitoring stations representative of the city as urban traffic (Boncompagni) and suburban and rural backgrounds (Cavaliere and Guido, respectively), the wind roses (Fig. 2) were obtained. Those concerning the urban site showed similar predominance (< 30%) of low northward speed winds (< 4 m/s) for both the years, but also a component from south and south-west, especially the former, associated with higher wind speeds, was observed. During 2019, two added provenience directions were also evident, from southeast and north-west with a higher percentage of stronger winds (2–3% of winds with > 4 m/s). The two background stations are positioned, respectively, in the eastern and western outermost edge of the Rome area and, at a different extent, can be influenced from the nearby urban center. At Cavaliere, the prevailing wind directions were from east and south-west in both years, but in 2019, weaker eastward winds were predominant, whereas the opposite happened in 2020. Since the city of Rome is located south-west of Cavaliere monitoring station, the transport process from the capital city could be possible in 2020, also due to the not negligible percentage of higher wind speeds (> 10% of winds > 4 m/s). At Guido station, the two prevailing wind directions from north and north-east were consistent in the 2 years. Eastward and southeastward winds were observed in 2019 (almost 6% of winds > 4 m/s from southeast), as well as west wind direction was also present in the 2020, but strengthened (almost 5% of winds > 4 m/s) in the 2019. The eastward wind direction in this case is coming from Rome. Overall, the winds recorded are mild breezes, conveying the air masses rather locally, and therefore, they can be considered contributing to pollutant dispersion, but not as primary factors affecting the air pollution conditions.

It can be concluded that the possible reductions of air pollutants such as NO_2_, NO, CO, and O_3_ observed in the compared periods were not highly determined by changes in meteorological conditions.

### Diurnal variations

#### Traffic urban stations

The study of diurnal variations of air pollutants can provide valuable information about the sources, transport, and chemical formation/destruction effects of such pollutants. In addition, the diurnal variations have a major influence on exposure levels at sites nominally exposed.

**Fig. 3:**
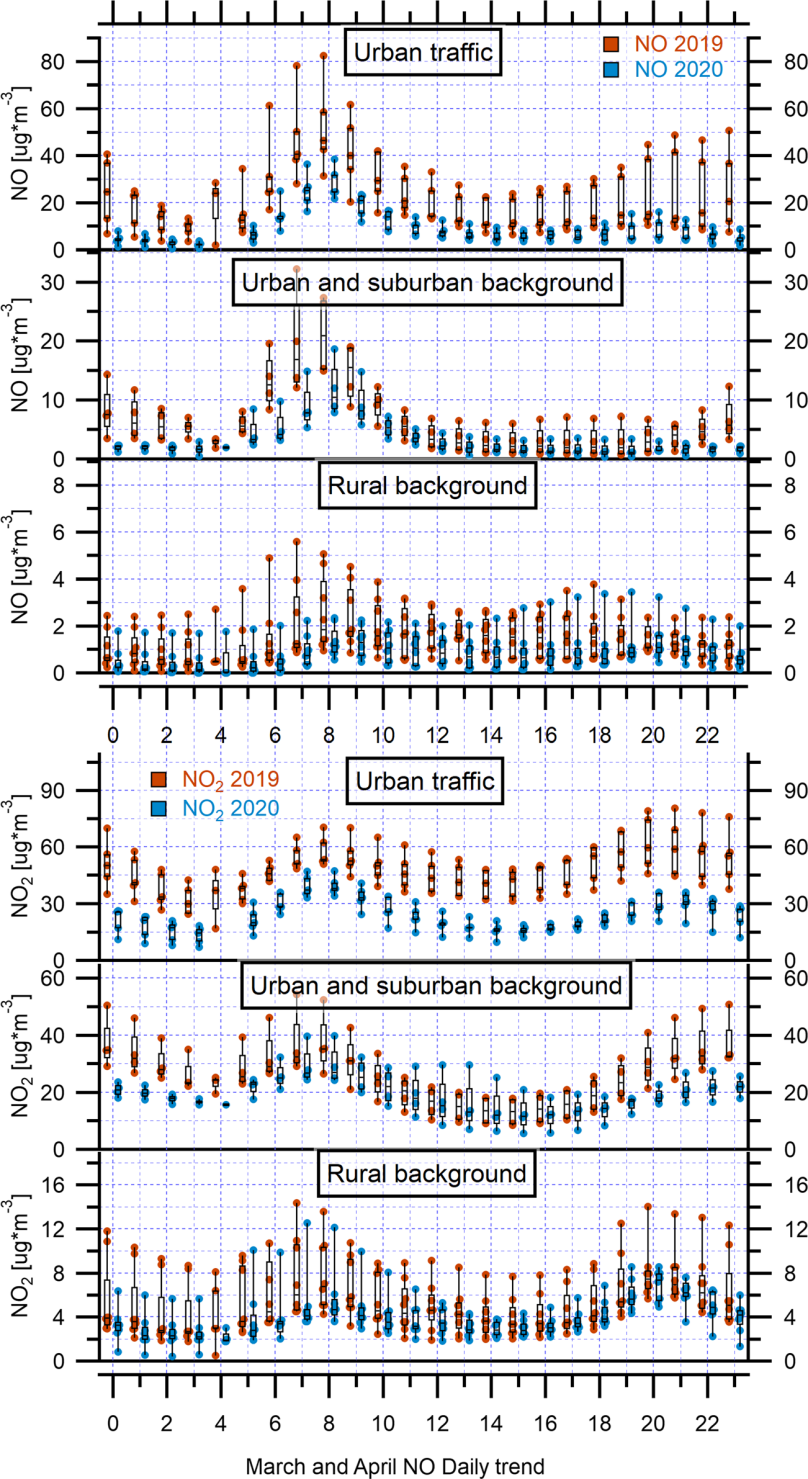
The box and whisker plots (with individual data points) of hourly mean NO_2_ and NO concentrations during March–April 2020 and March–April 2019 over urban traffic, urban and suburban background, and rural background monitoring stations in Rome and regional territory. The top and the bottom of each box represent 75th percentile and 25th percentile, respectively, and the upper and lower whiskers represent the outliers. The horizontal bar in each box represents the median

The hourly mean concentrations of NO_2_ and NO for March 2020 and April 2020 during the COVID-19 lockdown, and for March 2019 and April 2019 prior to the pandemic, averaging across six stations of Rome (Francia, Magna Grecia, Fermi, Tiburtina, Ciampino, and Civ. Via Roma), are shown in Fig. 3 and Fig. S7-S8. The two pollutants exhibited the typical urban daily pattern with a bimodal distribution at all sites. NO and NO_2_ began growing at 05:00, reaching a first peak between 07:00 and 09:00 (traffic rush hours) in the morning, with the second peak occurring between 20:00 and 22:00 at night. The second peak concentration of NO was significantly lower than the first peak. This could be attributed to less congested traffic from the traffic rush in the morning. The second peak concentration of NO_2_ was most of the time higher than the first peak. This could be caused by the reaction between NO and O_3_ to form NO_2_ at night in absence of solar radiation, resulting in its gradual accumulation (Song et al. 2011).

The comparison of hourly mean concentration values of March and April 2019 with March and April 2020 showed a clear declining trend in NO and NO_2_ levels. Furthermore, lower concentrations were observed for NO (Fig. 3 and Fig. S8), which, being directly emitted from fossil fuel processes, could be more sensitive to the lockdown period and, thus, to transport restrictions. Thus, the decline of NO_2_ and NO pollutants is attributable to the reduction of non-essential vehicles and combustion activities in industrial and commercial sites during the lockdown period.

**Fig. 4:**
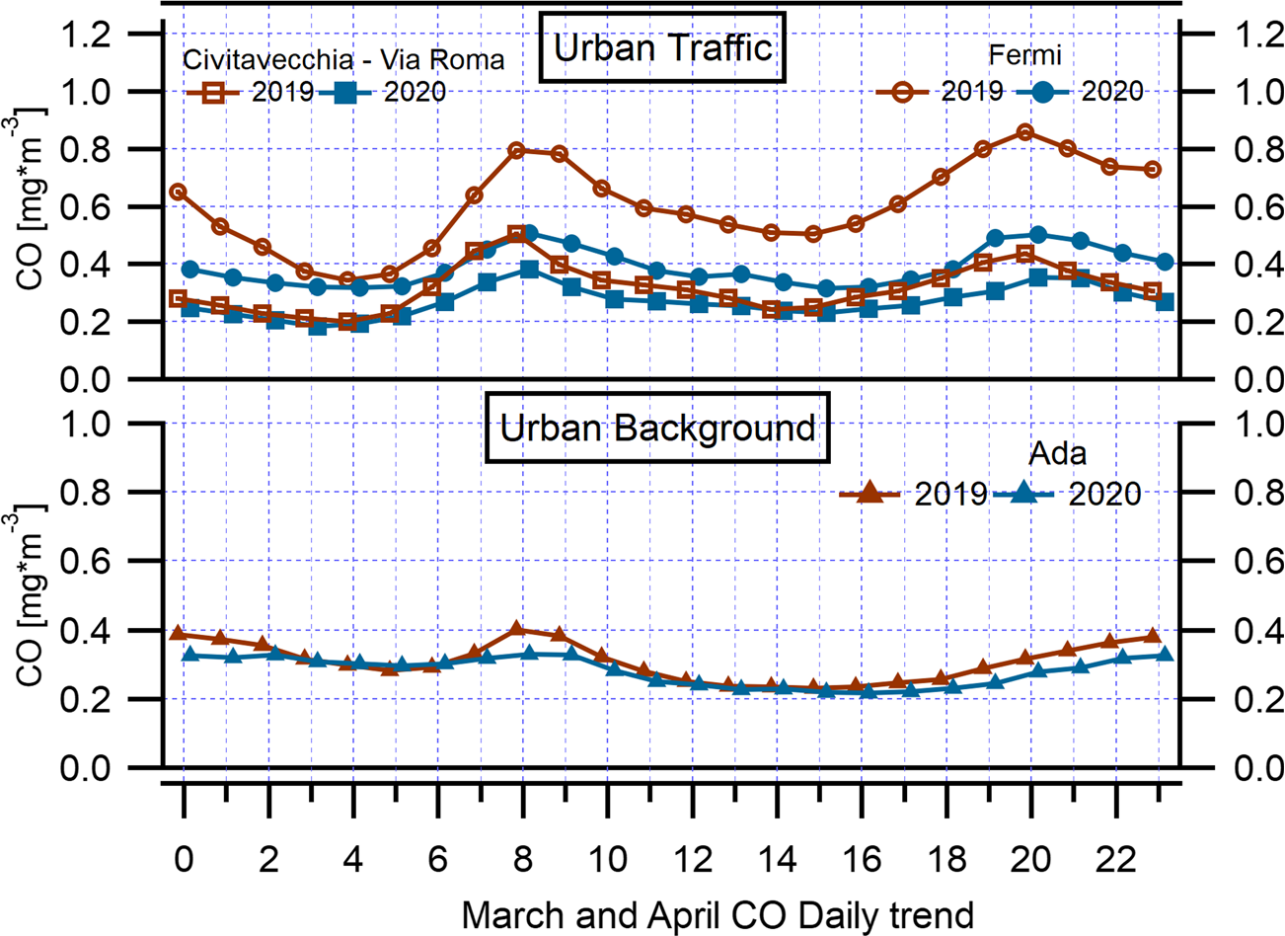
Diurnal variations of the hourly mean CO concentrations during March–April 2020 and March–April 2019 over urban traffic and urban background monitoring stations in Rome and regional territory

As for NO_2_ and NO trends, CO has a typical daily cycle linked to road traffic at two monitoring urban traffic stations (Fermi, Civ. Via Roma) in the Rome network (Fig. 4 and Fig. S9), where this atmospheric pollutant was measured, with two daily peaks corresponding to the morning and evening rush hours (08:00–09:00 and 20:00–21:00). According to the European Environmental Agency (2019), the primary sources of CO in Europe are from commercial, household, and institutional activities, accounting for 50% of the emissions, and from the road transport sector (19%). This suggests that implemented restrictions on transportation, agricultural, and industrial activities during the COVID-19 pandemic could lead to decreased CO levels. The hourly comparison of mean concentration values of March and April 2019 with March and April 2020 shows a less pronounced declining trend in CO levels compared to NO and NO_2_ trends, highlighting the contribution of the limited vehicular emissions and the reduction in the use of household heating to the CO concentration.

#### Background stations

Totally eight monitoring stations of the ARPA network were chosen for the assessment of the effectiveness of restrictions in lowering air pollution, and as a ground reference to be considered along with satellite measurements. As first approach, a “typical lockdown day” was obtained averaging the data collected during the month of March and April 2020 on an hourly basis, then for comparison, the same was also applied to the corresponding periods of the year 2019. From the diurnal NO_2_ and CO cycles, the typical “rush hour” peaks in the early morning and during the evening were evident, particularly for urban and suburban backgrounds plots (Fig. 3, Fig. 4, Fig. S10-S11 and S13 in Supplementary Material). The decrease due to lockdown can be clearly recognized in Fig. 3 relative to the average hourly values for each type of station over the whole bimonthly period of the 2 years. The statistical distribution is represented by the whisker boxplot in which data dispersion around the median value is also evident. In the NO_2_ plot of urban backgrounds, the decrease in 2020 with respect to 2019 is apparent in the evening-night hours, starting from 18:00, overnight, and in the first part of the morning until 10:00. During the rest of the day, there is no univoque behavior, and in the case of Ada station, the 2019 data are lower than 2020 (Fig. S10). At these sites, NO values have the sharpest decrease when comparing the 2 years from 06:00 to 08:00 in the morning in coincidence with the traffic rush hour. Among rural stations, Guido shows the highest values, which are evident outliers in the distribution obtained considering all the sites (Fig. 3). At this site, NO_2_ concentrations during the 2 years are rather similar in the early to the late morning (from 05:00 to 10:00–11:00), whereas for NO, the opposite happens with a more significant reduction from 05:00 to 10:00–11:00.

The diurnal cycles, reported in Figs. S10-S13, allow the comparison of the pollutant trends also according to the single station. The urban and suburban background sites showed, as expected, higher concentrations with respect to the rural. A bimodal distribution was observed during March 2020 for NO_2_, with a morning peak at 07:00–08:00 and an almost equal peak in the evening (at 22:00) at all the backgrounds. If we take into consideration the same period of the previous year, the peaks followed the same bimodal trend, but the concentration values were higher at Preneste and at Guglielmi. April 2019 showed a bimodal distribution as well, with peaks at the same hours, roughly corresponding to the “rush hour,” whereas during April 2020, the bimodal distribution disappeared. The diurnal cycles of NO showed an unimodal distribution during March 2019 and 2020 with a unique 08:00 o’clock peak at all the sites examined. The trend further flattened during April 2020 with lower morning peaks at 07:00 with respect to April 2019.

The same diurnal variations can be represented graphically for rural background sites. Among them, Guido is the nearest to the city of Rome (about 18 km from the city center), and it is affected by the proximity to the metropolitan area as shown by the higher values of the pollutants investigated, if compared to the other rural background sites.

The bimodal distribution is also characteristic of NO_2_ at rural sites during March 2020; the trend became unimodal in April 2020 as already remarked for the urban and suburban backgrounds. An evening peak was observed at 20:00 in Allumiere and in Leonessa. In these two locations, the evening peak is higher than in Guido and the peaks are almost equal in the 2 years. The plots of the month of March 2019 are rather similar for the 2 years and, as will be explained later, the difference seems to be negligible. Extending the comparison to April, an unimodal distribution was seen in 2020, whereas a bimodal trend characterized April 2019. A slight decrease in concentrations was observed during the month of April, particularly in the morning peaks of Guido and Acquapendente.

Very low NO values were measured at these sites, in many cases close or lower than the detection limit of the analyzer; therefore, the complete cycles are not reported and the observations are limited to the valid values. Evening peak values at 20:00–21:00 were observed in Allumiere in March 2019 and 2020. To a lesser extent, this was also evident in Leonessa, and likely due to domestic heating as proposed in a previous study (Petracchini et al. 2017) and also suggested by the decrease in April. The two sites are located in hill-mountainous areas where biomass combustion is still widely used, particularly during the evenings. The evening peaks were observed at Allumiere both for NO and NO_2_ at the same time, but NO concentration was higher in 2020 than in 2019.

NO_2_ and NO concentrations were also low at A. Liberti station and comparable with those found in other rural stations during the whole study period. NO_2_ was characterized by two daily peaks, while NO concentrations showed less diurnal variability during the same periods, devoiding the evening second peak. The comparison of hourly mean concentration values of March and April 2019 with March and April 2020 showed a clear declining trend in NO and NO_2_ levels. Indeed, significant reductions were also observed for NO levels (Fig. S16), showing a second peak due to anthropogenic activities such as the use of household heating (the heating season starts on the first of November and ends at the fifteenth of April).

Another primary pollutant which can provide evidence of the effectiveness of lockdown impact on pollution reduction is CO, but its measure is not foreseen at rural sites in monitoring networks. CO was measured only at Ada site (Fig. 4 and Fig. S13), where, as already seen for NO_2_, its distribution was bimodal, with maxima in the morning and in the evening. In 2020, the peak values were lower with respect to 2019.

**Fig. 5:**
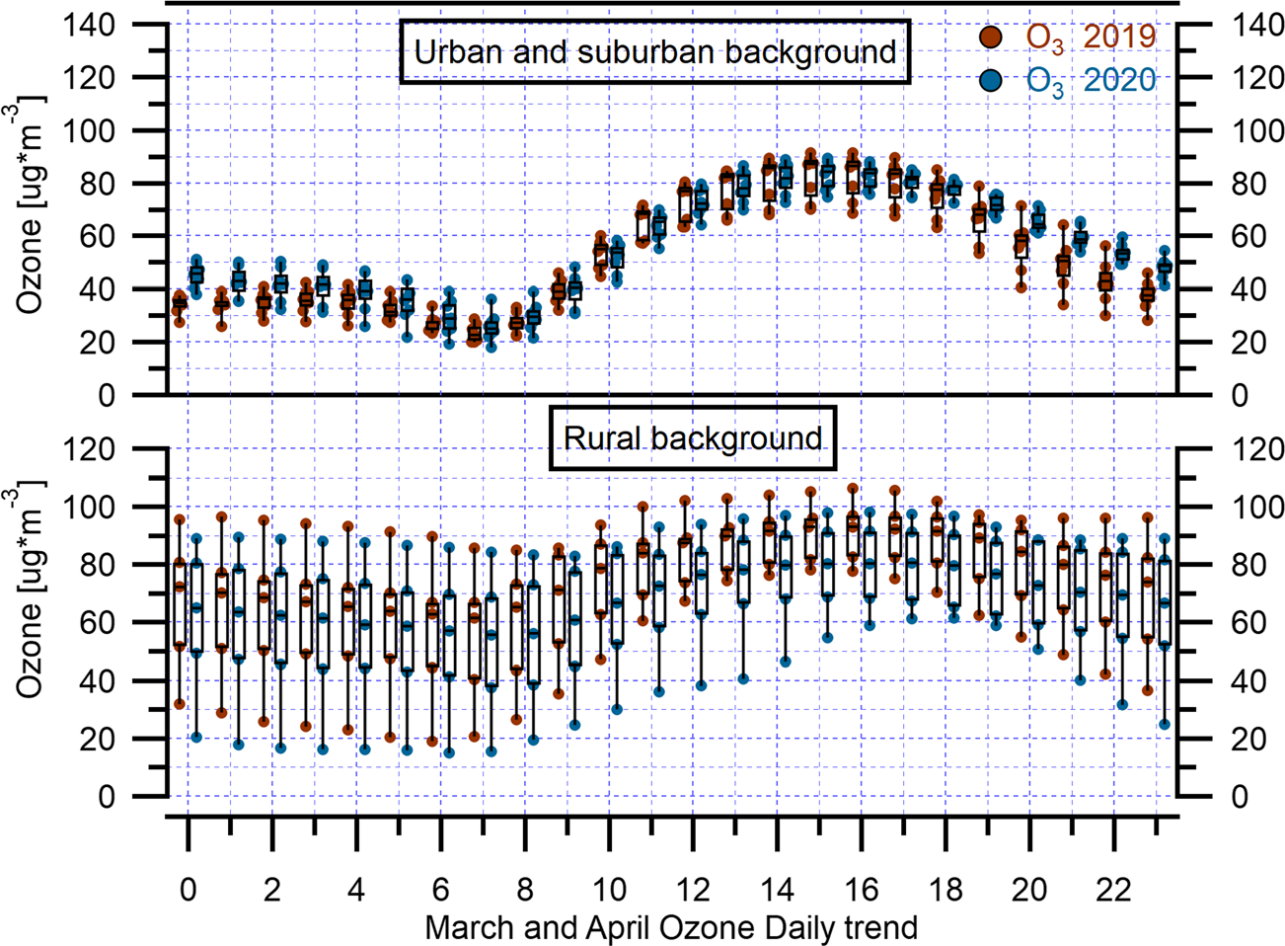
The box and whisker plots (with individual data points) of hourly mean O_3_ concentrations during March–April 2020 and March–April 2019 over urban and suburban background, and rural background monitoring stations in Rome and regional territory. The top and the bottom of each box represent 75th percentile and 25th percentile, respectively, and the upper and lower whiskers represent the outliers. The horizontal bar in each box represents the median

In general, the early spring period, during which the lockdown was enforced in the area under exam, is characterized by still slow O_3_ photochemistry due to the mild solar radiation, gradually increasing during the season. The diurnal cycles of urban and suburban backgrounds (Fig. 5 and Fig. S14) and rural sites (Fig. S15) look rather different, since the former shows an increasing trend with maxima at 15:00–16:00 in the afternoon whereas the latter exhibits a less pronounced trend, and in the case of Allumiere, a rather flat plot was obtained. An exception to this common trend among the rural backgrounds is represented by Guido station which is classified as rural, but is rather close to the city. The trend for this station during 2019 looks much more like urban and suburban backgrounds and shows the same modulation with afternoon growth, but appears somehow smoothened during 2020 (Fig. S15). Ozone concentration value at this site is lower with respect to the preceding year (Fig. S5 and Fig. S6), showing minimum daily average values in the periods 26–28 March 2020 and 16–22 April 2020 when a gradual decline was observed. The trends described were also found at Cavaliere station, where, in general, O_3_ mixing ratio during 2020 was similar to the previous year. The two periods were characterized, as reported in the section on meteorological data, by low temperature, and the days 20–22 April by low solar radiation (Fig. S3 and Fig. S4). Thus, the gradual ozone decrease in April is likely due to the delayed effect of the decrease in NO_x_ precursors, in combination with meteorological conditions unfavorable to ozone formation.

At A. Liberti station, the diurnal cycle of O_3_ consisted of nighttime low concentrations and daytime high concentrations, reaching a single broad peak between 15:00 and 16:00 h, lasting several hours (Fig. S16). This pattern was opposite to those of NO and NO_2_, which indicated the photochemical conversion from NO_x_ to O_3_ within NO_x_-O_3_-cycle during daylight hours. The hourly comparison of mean concentration values of March and April 2019 with March and April 2020 showed a declining trend in O_3_ concentrations. This diurnal variation in this station suggested that O_3_ concentrations have been reduced during the lockdown period in response to a decrease in ozone precursor emissions, as NO_x_, occurring in rural areas (Jin et al. 2020; Simon et al. 2015). Meteorological conditions, such as temperature, could affect the O_3_ trends but, as discussed previously, the meteorological data were almost similar prior to and during the lockdown periods, not influencing changes in air pollutant concentrations. However, at A. Liberti station ozone levels were lower than those measured in ARPA rural stations for both years (Fig. 5), especially during the first morning hours (between 0:00 and 06:00). In addition, during the period March–April 2020, the O_3_ decrease was more pronounced in comparison to ARPA rural measurements. These differences of O_3_ variation pattern between ARPA and A. Liberti stations could be due to the location of the latter placed in a transitional zone, with features of both non-consolidated urban and rural zones. A more detailed evaluation of these measurements including VOC observations, atmospheric transport, and dispersion dynamics is needed to obtain more robust conclusions on chemical processes affecting O_3_ levels.

Overall, it can be concluded that the possible observed variations of air pollutants such as NO_2_, NO, CO, and O_3_ in the compared periods (April 2020 vs. April 2019, and March 2020 vs. March 2019) were not highly determined by changes in meteorological conditions, but rather due to the lockdown restrictions. This is supported by observed variations in NO_2_ emissions (Table S1-S5) and by the statistical significance assessment performed and reported later.

### Monthly variations

#### Urban traffic stations

**Fig. 6:**
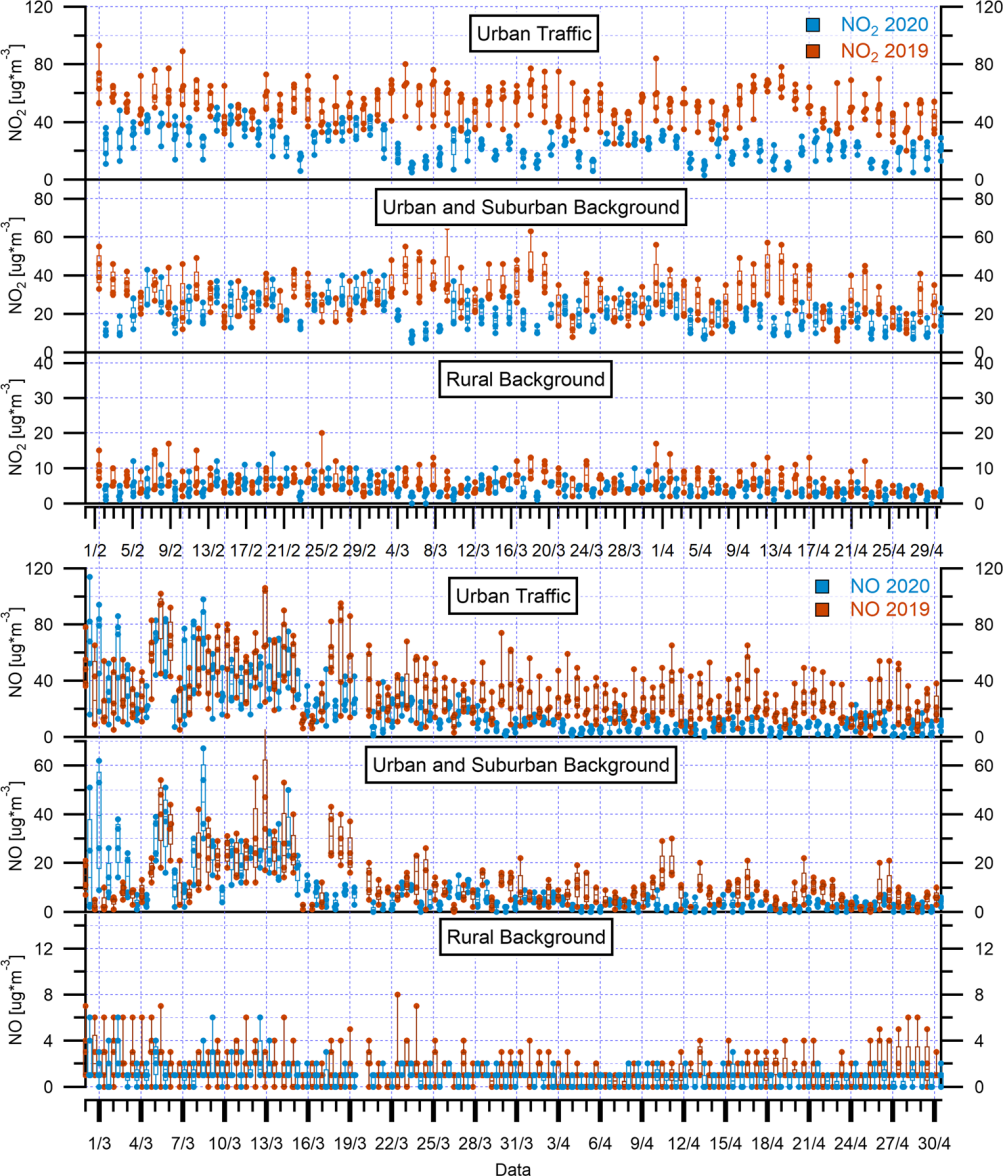
The box and whisker plots (with individual data points) of daily mean NO_2_ and NO concentrations during March–April 2020 and March–April 2019 over urban traffic, urban and suburban background, and rural background monitoring stations in Rome and regional territory. The top and the bottom of each box represent 75th percentile and 25th percentile, respectively, and the upper and lower whiskers represent the outliers. The horizontal bar in each box represents the median

Air pollution improvements were observed considering decreases in urban NO and NO_2_ levels of urban air pollutants measured during March and April 2020 in comparison to the same periods before the COVID-19 pandemic (March and April 2019) (Table 3, Table 4, and Fig. 6). Both NO and NO_2_ exhibited distinct and significant temporal variations with lower concentrations in March and April 2020 than in March and April 2019 in all urban traffic stations. The decline level ranged from − 34 to − 76% and from − 30 to − 65% for NO and NO_2_, respectively. In particular, maximum decline in mean monthly NO_2_ concentrations was observed in Civ. Via Roma urban traffic station, as also shown by the *t*-test results (*p* = 9.61*10^−152^ in March; *p* = 5.78*10^−142^ in April) followed by Francia station (*p* = 2.34*10^−128^ in March; *p* = 2.94*10^−180^ in April). Maximum decline in mean monthly NO concentrations was also observed in Francia station (*p* = 2.60*10^−57^ in March; *p* = 7.01*10^−80^ in April) followed by Fermi station (*p* = 1.22*10^−46^ in March; *p* = 1.91*10^−40^ in April). As said before, the road transport sector is the main source of NO_x_ emissions in Europe: in Rome, road transport corresponds to more than 50% of total NO_2_ emissions, as previously shown in Table S1. Similar reductions in air pollution have been observed in Italy and in other regions of the world (Gualtieri et al. 2020; Muhammad et al. 2020; Nakada and Urban 2020; Sicard et al. 2020; Tobías et al. 2020).

**Table 3:** NO_2_ reductions during lockdown March and April 2020, reported by surface monitoring stations, comparing the same periods to those in 2019

Monitoring stations		Mean NO_2_ March 2019 (μg/m^3^)	Mean NO_2_ March 2020 (μg/m^3^)	Reduction compared to March 2019 (%)	Mean NO_2_ April 2019 (μg/m^3^)	Mean NO_2_ April 2020 (μg/m^3^)	Reduction compared to April 2019 (%)	Mean lockdown reduction (%)
	Francia (UT)^a^	55.4	27.4	− 50	50.6	19.7	− 61	− 56
	Magna Grecia (UT)	43.0	30.0	− 30	38.5	23.1	− 40	− 35
	Fermi (UT)	64.7	33.2	− 49	60.6	21.4	− 65	− 57
	Tiburtina (UT)	58.5	32.0	− 45	53.0	20.6	− 61	− 53
	Ciampino (UT)	30.7	20.3	− 34	24.8	13.2	− 47	− 40
	CV. Via Roma (UT)	46.5	19.0	− 59	44.7	15.6	− 65	− 62
	Preneste (UB)^b^	41.9	27.7	− 34	38.2	18.3	− 52	− 43
	Ada (UB)	27.0	22.7	− 16	22.2	23.3	n. a.*	− 16
	F. Guglielmi (UB)	29.5	19.4	− 34	29.5	14.5	− 51	− 43
	Cavaliere (SB)^c^	25.7	19.2	− 26	19.7	13.6	− 31	− 28
	Allumiere (RB)^d^	5.5	4.6	− 17	4.4	4.0	− 10	− 13
	Acquapendente (RB)	5.8	4.5	− 22	4.7	3.1	− 34	− 28
	Guido (RB)	8.9	8.0	− 11	9.4	5.5	− 42	− 26
	Leonessa (RB)	4.6	3.9	− 14	3.7	3.2	− 14	− 14
	Montelibretti (RB)	11.7	5.0	− 57	9.7	3.7	− 62	− 60

^a^Urban traffic (UT)

^b^Urban background (UB)

^c^Suburban background (SB)

^d^Rural background (RB)

*Not applicable (not significant)

**Table 4:** NO reductions during lockdown March and April 2020, reported by surface monitoring stations, comparing the same periods to those in 2019

Monitoring stations	Mean NO March 2019 (μg/m^3^)	Mean NO March 2020 (μg/m^3^)	Reduction compared to March 2019 (%)	Mean NO April 2019 (μg/m^3^)	Mean NO April 2020 (μg/m^3^)	Reduction compared to April 2019 (%)	Mean lockdown reduction (%)
Francia (UT)^a^	42.8	16.1	− 62	36.1	8.7	− 76	− 69
Magna Grecia (UT)	17.8	11.7	− 34	14.7	7.6	− 49	− 41
Fermi (UT)	36.2	14.7	− 59	32.0	10.8	− 66	− 63
Tiburtina (UT)	24.7	12.3	− 50	19.4	6.7	− 65	− 58
Ciampino (UT)	14.4	6.0	− 58	10.6	3.9	− 63	− 61
Civ. Via Roma (UT) (UT)	14.9	8.0	− 46	13.9	7.5	− 46	− 46
Ada (UB)^b^	10.5	6.2	− 41	8	4.5	− 43	− 42
F. Guglielmi (UB)	8.6	4.6	− 46	7.2	2.8	− 61	− 53
Cavaliere (SB)^c^	7.9	4.3	− 46	5.7	2.8	− 50	− 48
Guido (RB)^d^	2.7	1.7	− 38	2.6	1.7	− 36	− 37
Montelibretti (RB)	3.2	2.5	− 23	3.1	2.1	− 31	− 27

^a^Urban traffic (UT)

^b^Urban background (UB)

^c^Suburban background (SB)

^d^Rural background (RB)

**Fig. 7:**
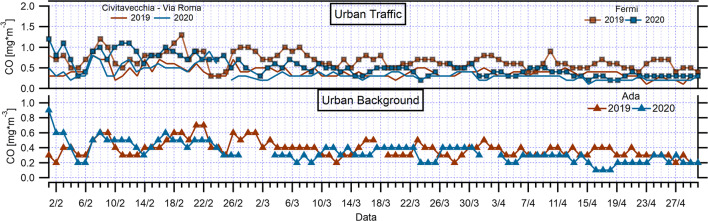
Temporal variations of daily mean CO concentrations during March–April 2020 and March–April 2019 over urban traffic and urban background monitoring stations in Rome and regional territory

Significant changes were also observed in CO levels (Table 5 and Fig. 7). In particular, maximum decline in mean monthly CO concentrations was observed in Fermi urban traffic station (*p* = 8.97*10^−45^ in March; *p* = 1.12*10^−89^ in April) followed by Civ. Via Roma station (*p* = 1.44*10^−11^ in March; *p* = 3.82*10^−34^ in April). The lower magnitude of the decrease in CO compared to NO_2_ reflects the different source contributions and lifetimes of the two pollutants. Atmospheric CO lifetime is generally longer than 30 days and, thus, its concentrations could be affected by long-range transport of pollution. Because atmospheric NO_2_ lifetime is less than a day in urban areas (a few hours in Rome during spring and summer periods) (Verstraeten et al. 2018), its concentrations are closely related to local sources much more directly than CO. There are also differences in emission sources such as the transport sector for CO (19%) and NO_2_ (39%), as said previously, not providing similarity in decrease of two pollutants in the time period during the COVID-19 pandemic.

**Table 5:** CO reductions during lockdown March and April 2020, reported by surface monitoring stations, comparing the same periods to those in 2019

Monitoring stations	Mean CO March 2019 (mg/m^3^)	Mean CO March 2020 (mg/m^3^)	Reduction compared to March 2019 (%)	Mean CO April 2019 (mg/m^3^)	Mean CO April 2020 (mg/m^3^)	Reduction compared to April 2019 (%)	Mean lockdown reduction (%)
Fermi (UT)^a^	0.7	0.5	− 31	0.6	0.3	− 44	− 37
Civ. Via Roma (UT)	0.4	0.3	− 16	0.3	0.2	− 28	− 22
Ada (UB)^b^	0.4	0.3	− 11	0.3	0.2	− 27	− 19

^a^Urban traffic (UT)

^b^Urban background (UB)

Since meteorological conditions did not affect changes in pollutant concentrations, these results highlighted the improvement of air pollution for NO, NO_2_, and CO due to lockdown restrictions which led to reduced primary pollutant emissions in Rome (for instance, a − 67% average hourly car passes, as previously shown in Table S2).

#### Background stations

The months of March and April 2020 were differently impacted by the lockdown restrictions, since, as already reported, there was a gradual implementation starting from 10 March, and the concentrations at backgrounds declined accordingly. Applying the two sample *t*-test, it was found that in almost all the urban and suburban background sites, the reduction was significant. During both the months of March and April 2020, with respect to the same periods of 2019, the reduction of NO_2_ was significant at the totality of the stations (*p* < 0.05) with the exception of the station of Ada (*p* = 0.122 in April) in the urban area. The values of NO_2_ mixing ratios had a substantial decrease, among these sites, as reported in Table 3. NO decrease was significant at the totality of the urban and suburban background stations examined during the month of March and April (*p* < 0.05). The highest decrease of NO concentration was calculated for Fiumicino Guglielmi, followed by the stations of Cavaliere and Ada as reported in Table 4, during April 2020 in comparison to April 2019, when the reduction of both the pollutants became larger. CO concentration reduction measured at Ada station was significant both in March and in April 2020 (*p* < 0.05) (Table 5).

Moving to rural areas, NO_2_ decrease significance was successfully assessed (*p* < 0.05) at all of the stations. As expected, NO mixing ratios at these locations were normally lower than in urban or suburban backgrounds. In April 2020, values below the detection limit were measured almost everywhere except for Guido station. Despite the low number of values above the L.O.D., performing the *t*-test on the data available a significant reduction (*p* < 0.05) was observed at all of the stations, except for Leonessa, during both of the months.

Significant improvements of air pollution were also observed in A. Liberti Station during March and April 2020, considering the percentage of variation in NO_2_ and NO concentrations, comparing the same periods before the pandemic (March and April 2019) (Table 3 and Table 4). Both NO_2_ and NO exhibit significant reductions in March (*p* = 7.19*10^−87^ and *p* = 2.11*10^−17^, respectively) and April 2020 (*p* = 3.60*10^−122^ and *p* = 1.27*10^−25^, respectively). Unlike other rural sites of the ARPA network, NO_2_ levels were reduced much more than NO. Background sites which, in most of the cases are sited in green areas, are particularly subject to photochemical pollution due to the peculiar conditions which lead to the formation of ozone and other species involved in this complex of reactions. A detailed analysis goes beyond the scope of this study; anyway some observations can be reported to better understand the effects of this involuntary and extreme traffic control measure exercise. The effects of lockdown on NO_2_ reduction were easily assessed locally, whereas the global impacts which affect the O_3_ concentration are subject to transport, weather conditions, and related to site-specific characteristics, therefore not a priori predictable and different in urban and rural areas.

**Fig. 8:**
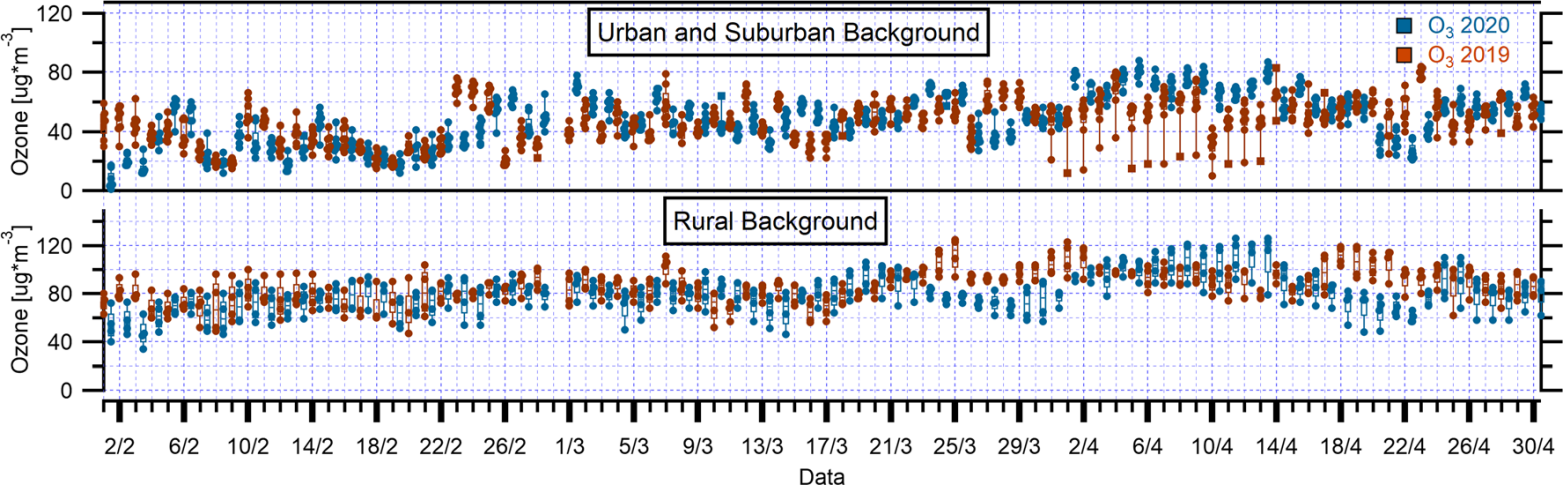
The box and whisker plots (with individual data points) of daily mean O_3_ concentrations during March–April 2020 and March–April 2019 over urban and suburban background, and rural background monitoring stations in Rome and regional territory. The top and the bottom of each box represent 75th percentile and 25th percentile, respectively, and the upper and lower whiskers represent the outliers. The horizontal bar in each box represents the median

Applying the two sample *t*-test to assess the significance of the variation in O_3_ concentrations with respect to the whole March and April period of the previous years, a significant change was assessed at the totality of the stations (*p* < 0.05) except for Malagrotta (*p* = 0.089). O_3_ levels recorded site-to-site variability showing a slight average increase in urban and suburban background stations and a slight average decrease in rural background stations (Table 6, Table S6, and Fig. 8) during the lockdown period. The sharp decrease in NO_2_ concentrations in the rural site could lead to a decrease in ozone, since atmospheric chemistry is under NO_x_-limited regime whereas for urban areas the opposite happened, as previously demonstrated (Sicard et al. 2013; Simon et al. 2015; Witte et al. 2011). These results are consistent with recent findings observed in Italy and in other regions of the world (Gualtieri et al. 2020; Sharma et al. 2020; Sicard et al. 2020; Venter et al. 2020; Xu et al. 2020a, 2020b; Zhao et al. 2020).

**Table 6:** O_3_ changes during lockdown period averaged over March–April 2020, reported by surface monitoring stations, comparing the same periods to those in 2019

Monitoring stations	Mean O_3_ 2019 (μg/m^3^)	Mean O_3_ 2020 (μg/m^3^)	Changes (%)
UB^a^	51.1	54.7	7
SB^b^	49.7	57.2	15
RB^c^	75.5	67.1	− 11

^a^Urban background (UB)

^b^Suburban background (SB)

^c^Rural background (RB)

### TROPOMI tropospheric NO_2_ VCD

The Royal Netherlands Meteorological Institute (KNMI) obtained a tropospheric NO_2_ reduction of − 49% in the city of Rome from the averaged TROPOMI products from 13 March until 13 April 2020 compared with the same period of 2019 screened of the weather conditions (processed by KNMI/ESA, https://directory.eoportal.org/web/eoportal/satellite-missions/c-missions/copernicus-sentinel-5p). This drastic NO_2_ reduction drives us to analyze the daily tropospheric NO2 VCD in the pixels including the air quality stations in the city of Rome. The selected TROPOMI maps are 55 images acquired in the period from 10 March till 3 May 2019 and 55 images for the same period of 2020. The restriction to cloud-free observations is guaranteed by applying the quality assurance (qa) condition qa > 0.75 (Verhoelst et al. 2020; van Geffen et al. 2019). Besides, the detected NO_2_ VCD is verified to be greater than the precision of the tropospheric NO_2_ product provided by the field nitrogendioxide_tropospheric_column_precision which ensures the reliability of the tropospheric NO_2_ also in case of very low-level concentration like retrieved from the chemical analysis in rural background sites during the lockdown. At the end of the screenings, the actual amount of daily TROPOMI maps suitable to extract the VCD related to each monitoring station are 30 (2019) and 38 (2020) in Rome, and 37 (2019) and 42 (2020) in the rural background.

**Fig. 9:**
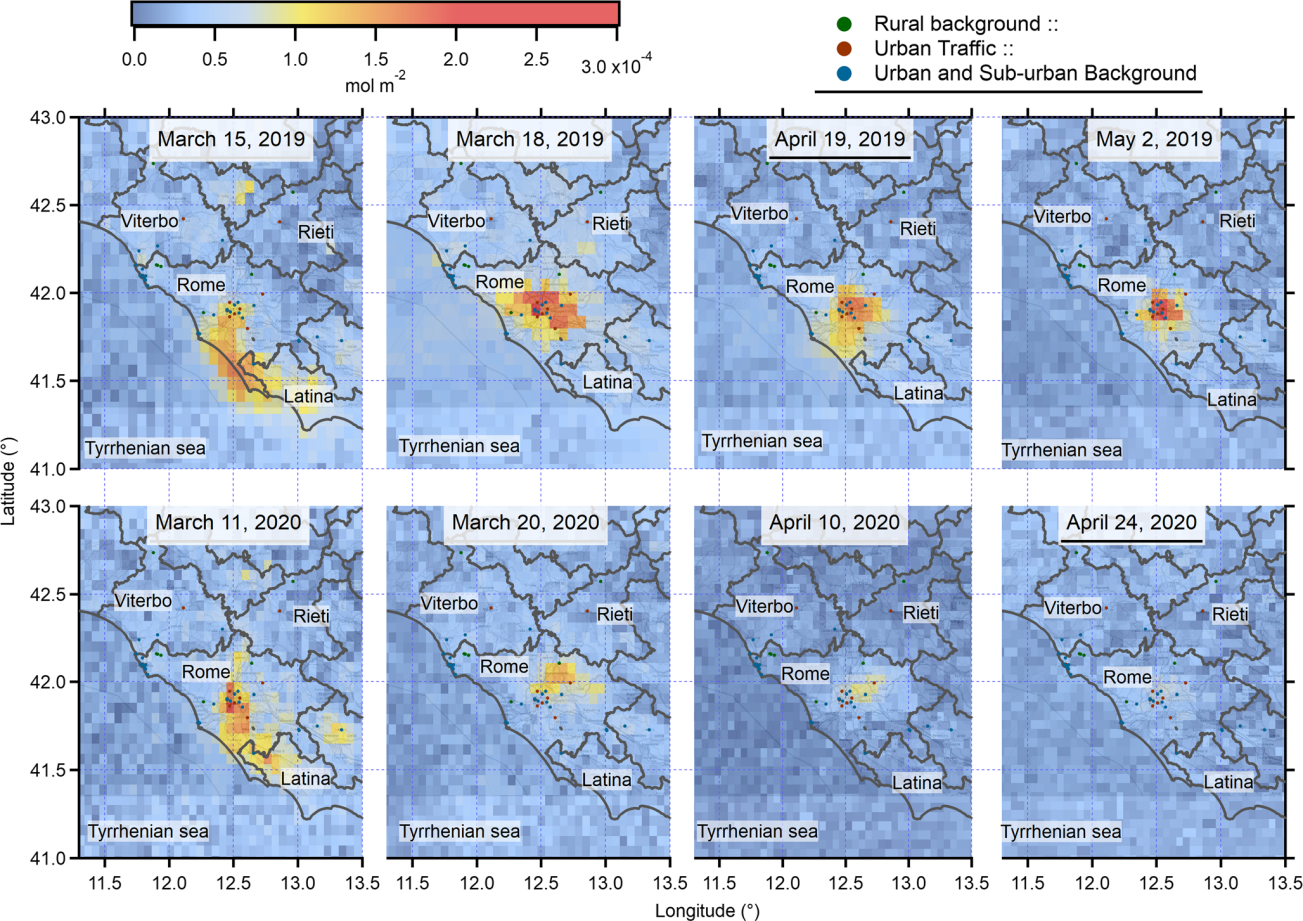
Examples of spatial distribution of the TROPOMI tropospheric NO_2_ VCD in the study area before (10 March–3 May 2019) and during (10 March–3 May 2020) the lockdown. Above, from the left to the right the maps of 2019/03/15; 2019/04/18; 2019/04/19; 2019/05/02. Below, from the left to the right 2020/03/11; 2020/03/20; 2020/04/10; 2020/04/24

Figure 9 shows examples of the daily spatial distribution of the tropospheric NO_2_ VCD (mol m^−2^ unit) during the Italian lockdown started on 10 March and extended to 3 May 2020 and the same period of 2019. The sensor detects localized enhancements of tropospheric NO_2_ on the hotspot of Rome at the scale of pixel size (red pixels). Above, the remotely sensed maps of the first row are the temporally ordered NO_2_ tropospheric VCD maps of 2019/03/15; 2019/04/18; 2019/04/19; and 2019/05/02. In the lockdown, a decrease of the NO_2_ levels is expected for the reduction of the amount of urban emission sources. Figure 9 reveals the mitigation of the tropospheric NO_2_ VCD during the Italian lockdown as retrieved from the NO_2_ surface concentration underlined in Fig. 6. From left to the right of the second row of Fig. 9, the NO_2_ hotspot gradually decreases during the significant reduction of the emission sources in the urban site. At the beginning of lockdown (2020/03/11), the tropospheric nitrogen dioxide is similar to the values of the previous year; afterward the tropospheric NO_2_ decreases until the disappearance of the hotspot in the map at the end of April 2020 (2020/04/24).

The analysis of the TROPOMI product focuses on the pixels where NO_2_ concentrations are measured at ground. In this way, the spatial representativeness of the NO_2_ VCD reduction can be discussed by the concurrent surface measurements provided by the 15 air quality stations enclosed in the study area. In order to fully understand the spatial and temporal distribution of the NO_2_ reduction from space, the remotely sensing data are classified according to the NO_2_ VCD level. Generally, the TROPOMI pixels in the urban site are characterized by high level of NO_2_ VCD which can be the result of a combination of different atmospheric environments such as traffic and urban background (see the Rome maps in Fig. 1). Thus from space, the highly polluted city under study appears as a combination of the six urban background (Arenula, Preneste, Ada, Bufalotta, Cipro, Cinecittà) and the four urban traffic (Francia, Magna Grecia, Fermi, Tiburtina) monitoring stations throughout the city of Rome. Regarding the NO_2_ low level, the pixels, that include the five rural background stations (Castel di Guido, Montelibretti, Leonessa, Allumiere, and Acquapendente), are considered representative of lower NO_2_ concentration in the study area following the results obtained from the chemical analysis.

During the period from 10 March to 3 May 2019, the NO_2_ high level was on average 9.35e^−05^ mol m^−2^ about 2.6 times higher than the NO_2_ observed in the pixel including the rural background stations 3.65e^−05^ mol m^−2^. During the lockdown, the averaged NO_2_ VCD in Rome was 5.29e^−05^ mol m^−2^ and in rural background was 3.02e^−05^ mol m^−2^ which means a decrease of ratio to 1.7 leading to achieving the NO_2_ level in urban site more similar to the level usually measured in rural background. This effect is the particular environmental condition of the lockdown where the difference between the urban site and the rural background sites appears, on average, substantially reduced. Besides, the tropospheric NO_2_ in the urban pixels belonging to the hotspot goes up to about 3.82e^−04^ mol m^−2^ in 2019 and does not exceed 1.74e^−04^ mol m^−2^ during the lockdown.

**Fig. 10:**
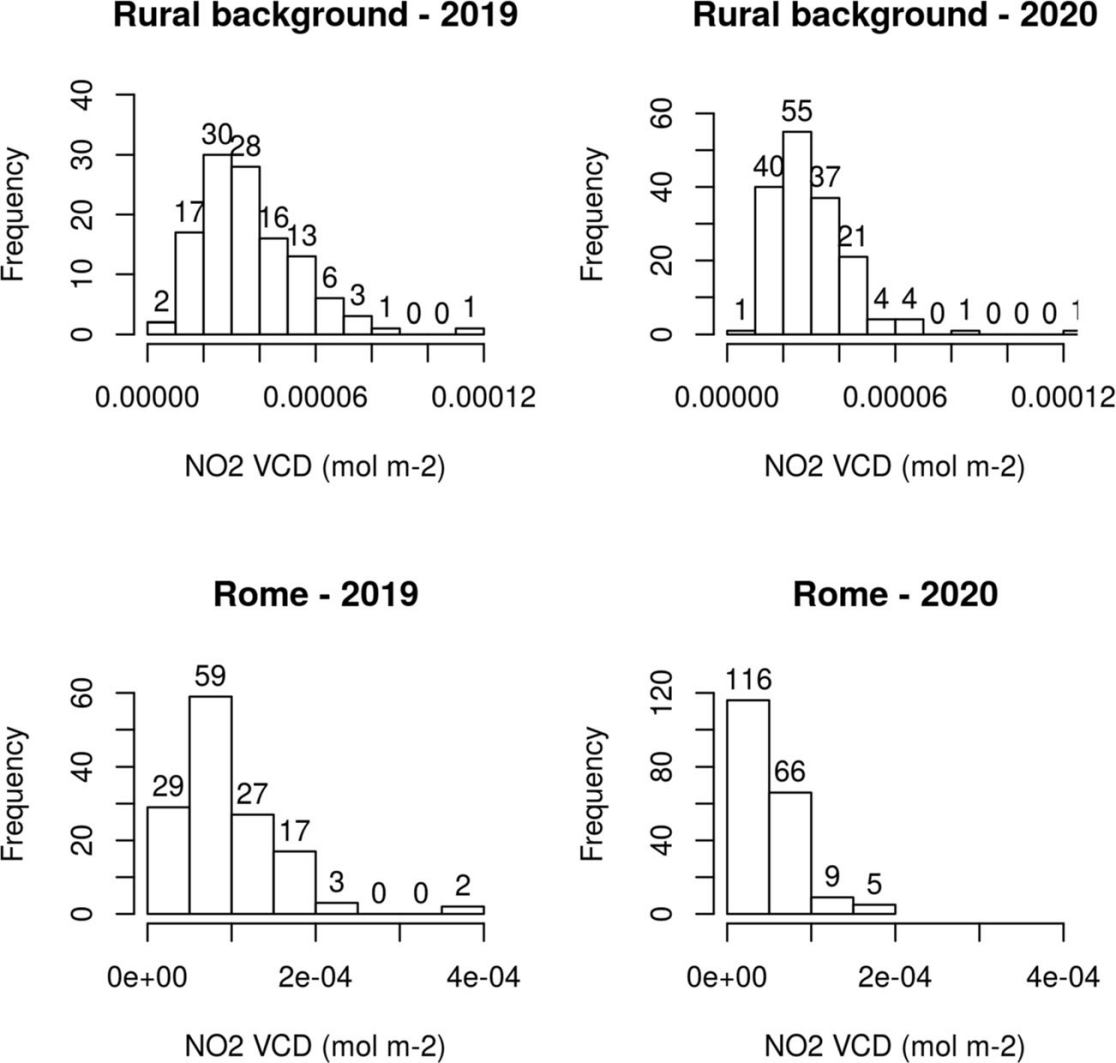
Frequency distribution of NO_2_ VCD in the pixels with the monitoring stations of rural background (above) and located in Rome (below) during the lockdown (March 10–May 3, 2020) and the corresponding period in 2019

Figure 10 shows the frequency distribution of the NO_2_ VCD for all the pixels including the monitoring stations in the rural background and in the city of Rome. During 2019, both the NO_2_ VCD distributions appear well-distributed and unimodal, becoming even more positively skewed distributed during the lockdown. Indeed, during the lockdown, the two distributions become light-tailed with an increase of pixel amount around the peaks especially in the urban site. In Rome, the peak moves from around 7.50 mol m^−2^ to 2.50e^−05^ mol m^−2^ close to the peak of rural background. This agrees with the previous findings of significant NO_2_ reduction in the urban site toward the decreasing of emission sources that explain the disappearance of the hotspot in the maps at the end of April 2020.

The reduction of the tropospheric NO_2_ VCD is evaluated by comparing the average of the pixel values extracted from the TROPOMI maps from 10 March to 3 May 2020 with respect to the average of the maps referred to the same period of the previous year. The reduction for pixels with all the surface stations is − 32%, whereas for urban pixels composed of urban and urban background stations is − 43% and for pixels with stations of rural background, a more slight reduction is observed (− 17%). This confirms the reduction retrieved by ESA in the urban site and also highlights the decreasing of NO_2_ VCD in the rural background. The NO_2_ VCD reduction of all the pixels within the urban site is comparable to the reductions of surface NO_2_ concentration obtained in the same time period (from 10 March to 3 May) in the air quality stations (traffic and urban background) included in the pixels (− 45%). Besides, the reduction in the surface stations of rural backgrounds (− 20%) obtained in the same period is similar to the VCD reduction obtained in low level (− 17%). These results point to the TROPOMI capability to represent the NO_2_ reduction also when concentrations are low.

The evaluation of the TROPOMI capability to detect the spatial and temporal variability of the NO_2_ VCD is performed with a comparison between the satellite NO_2_ products and the concurrent surface concentrations. The daily NO_2_ concentration in the rural background was obtained by averaging the five monitoring stations and the corresponding five TROPOMI pixels are averaged to retrieve the daily VCD. In Rome, the daily tropospheric NO_2_ VCD is obtained by averaging the pixels where one or more surface stations are located. The resulting NO_2_ VCD is compared with the concurrent surface concentration achieved by averaging the surface NO_2_ of all the urban background and traffic stations.

**Fig. 11:**
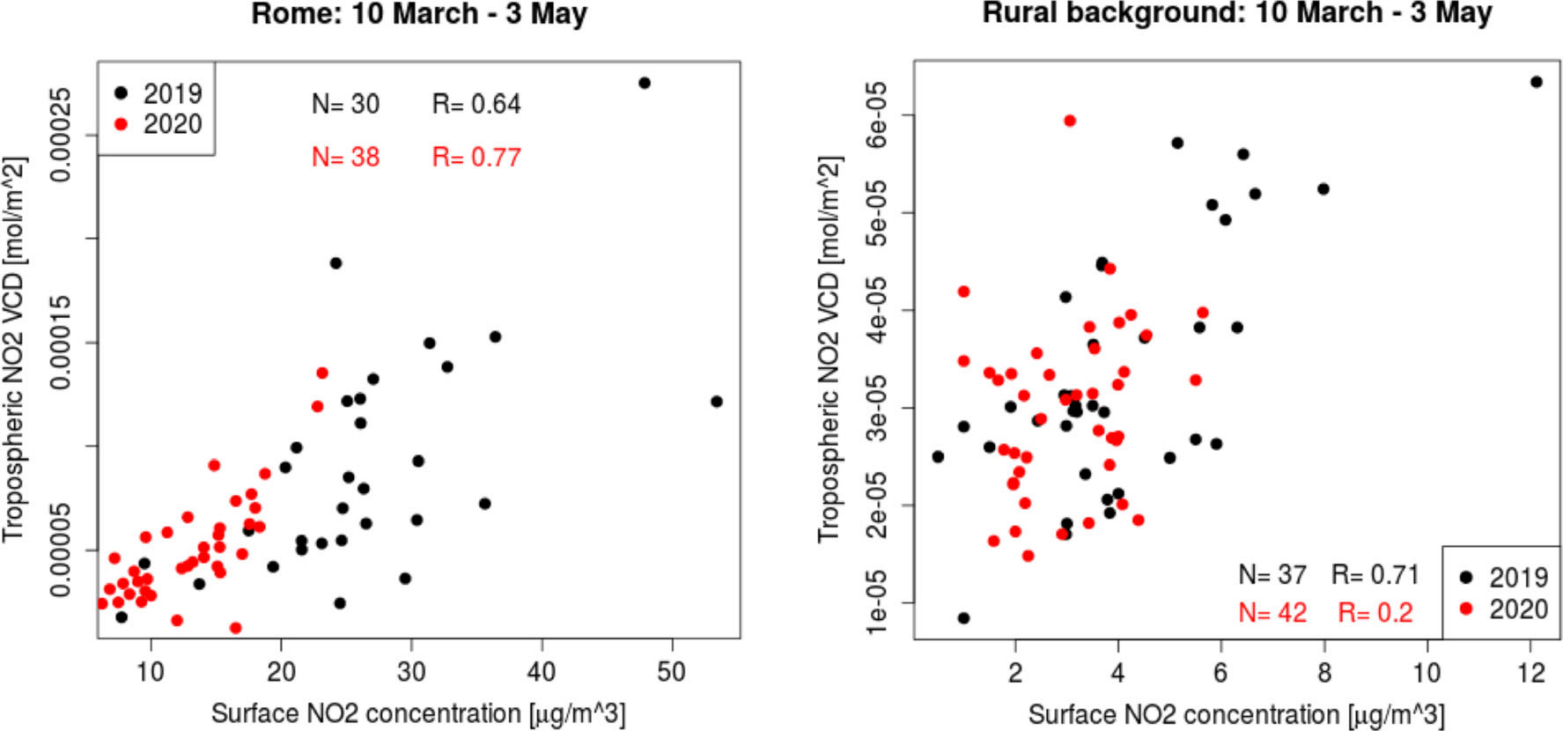
The daily averaged TROPOMI tropospheric NO_2_ column (VCD) vs. surface NO_2_ concentrations before, 10 March–3 May 2019, and during the Italian lockdown, 10 March–3 May 2020. In the urban site, the average is performed on the pixels including the surface monitoring stations

Figure 11 shows the scatterplot of the daily averaged tropospheric NO_2_ VCD vs. surface NO_2_ concentrations at monitoring stations during the lockdown, 10 March–3 May 2020, and in the same period of the previous year. In Rome, the good correlation is maintained in the high-level condition of 2019 with correlation Pearson coefficient of *R* = 0.64 (*p* = 2.088e^−15^) and in the lower level condition occurs during the lockdown with *R* = 0.77 (*p* < 2.2e^−16^). These results were consistent with previous studies carried out in a Canadian site characterized by significant NO_x_ emissions (Griffin et al. 2019). In rural background, good agreement is obtained in usual NO_2_ concentration attested by *R* = 0.71 (*p* = 1.426e^−13^), while large discrepancies appear between the satellite and surface data during the lockdown, with the assessment of correlation index *R* = 0.20 (*p* < 2.2e^−16^). The physical basis behind this discrepancy might be due to the very low-level observed NO_2_ that reaches the minimum achievable and detectable limit. In general, the retrieved correlations reveal the high capability of a coupled system composed of surface and satellite observations to monitor the daily variation of NO_2_ in the urban site and also in the surrounding rural background affected by local emission sources and possibly by the neighboring urban site.

In order to compare the satellite and surface NO_2_, the surface concentrations are converted in the tropospheric VCD (*VCDsurface*) expected to be dominated by the atmospheric boundary layer (ABL) contribution. In Dieudonné et al. (2013), the retrieved linear relationship between surface concentration and VCD in the ABL allows to account for the vertical NO_2_ gradient and for the variable mixing ratio in the ABL over polluted sites where large NO_x_ emissions occur. Besides, the relationship is useful when a pixel-by-pixel comparison is performed in a shorter time scale. Thus, their empirical equation to derive the *VCDsurface* taking into account the vertical gradients of NO_2_ concentration in the ABL is applied (Lorente et al. 2019; Dieudonné et al. 2013):

*VCDsurface* = *K*(0.244*h*(*C*_g_ − 1.38) + 0.184(*C*_g_ − 2.83)) (1)

*K* is the unit conversion factor obtained by Dieudonné et al. (2013), *h* is the boundary layer height provided by the European Centre for Medium-Range Weather Forecasts (ECMWF) at the time of acquisitions; *C*_g_ is the averaged surface concentration calculated for Rome and background sites concurrent to the TROPOMI acquisitions. In order to comply with the SI unit definitions, the *VCDsurface* is converted in mol m^−2^, as the TROPOMI unit, rather than in the commonly used unit molec cm^−2^ (van Geffen et al. 2019, page 42; Eskes et al. 2019, page 19). Dieudonné et al. (2013) highlight the capability of this relationship to relate the satellite observations to the surface concentrations over Paris and very likely also over other cities. A good agreement between the *VCDsurface* and the TROPOMI VCD is retrieved in the urban site before (*R* = 0.59, *p* = 7.69e^−10^) and during (*R* = 0.73, *p* = 1.512e^−10^) the lockdown whereas in the rural sites, the correlation decreases from *R* = 0.74 (*p* = 0.033) to *R* = 0.17 (*p* = 3.359e^−10^), respectively. These correlations are consistent with the correlations reported in Fig. 11.

**Fig. 12:**
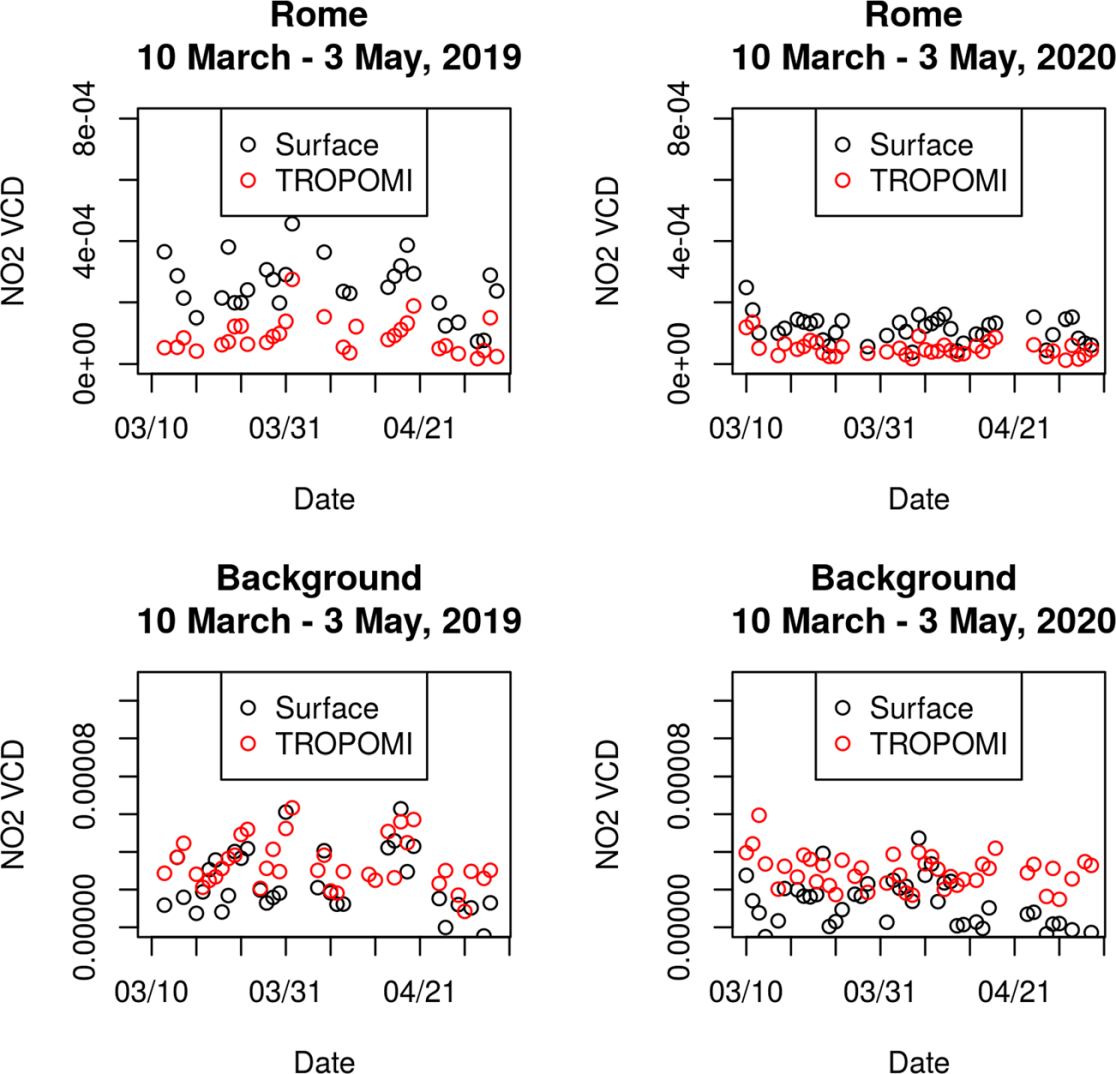
The temporal variability before (2019) and during (2020) the Italian lockdown of the surface (black points) and satellite (red points) NO_2_ VCD. The red points are the average of the pixels including the ground stations of each TROPOMI image acquired over Rome (high level) and the rural background (low level); the black points are the VCD obtained by Eq. (1) from the averaged concentrations of all the stations located in Rome and in rural backgrounds

Finally, the temporal variability of the satellite and surface NO_2_ VCD are evaluated for the whole period of lockdown and the same period of the previous year. Figure 12 shows the averaged NO_2_ *VCDsurface* for each day (black points) and the corresponding tropospheric VCD (red points) obtained by averaging the concurrent TROPOMI pixels including the monitoring stations. In high-level concentration, the satellite and surface products reveal the same pattern before and during the lockdown with an expected variability of satellite NO_2_ for the atmospheric inhomogeneity of the considered pixels. In low-level concentration, TROPOMI seems to overestimate the NO_2_ in 2019, preserving the general trend as in the case of the urban site in the same period, while during the lockdown, the data exhibit a noisy trend as expected for uncorrelated data as well as the minimum *VCDsurface* corresponding to the instrumental detection limit in agreement with the chemical analysis results.

The mean difference (*MD*) is used to evaluate the agreement between NO_2_ VCD retrieved from surface concentrations (*VCDsurface*) and that provided by the satellite (*VCDtropomi*):

2 $$ MD=\left(1/n\right)\ast {\sum}_{i=1,n}\left({VCDtropomi}_i-{VCDsurface}_i\ \right) $$

where *n* is the amount of data which corresponds to the TROPOMI images available after the screenings (rural background *n* = 37 in 2019 and *n* = 42 in 2020; urban site *n* = 30 in 2019 and *n* = 38 in 2020). A negative *MD* value attests that NO_2_ is underestimated from satellite respect to the surface concentration in high-level condition (*MD* = − 1.71*10^4^ mol m^−2^ in 2019 and *MD* = − 0.63*10^4^ mol m^−2^ in 2020) while a positive *MD* value attests an overestimation in low-level condition (*MD* = 0.10*10^4^ mol m^−2^ in 2019 and *MD* = 0.177*10^4^ mol m^−2^ in 2020). These results are in agreement with the *MD* changes from positive to negative under increasing NO_2_ concentration obtained from the comparison between TROPOMI and ground-based NO_2_ total columns reported in Ialongo et al. (2020). In Rome, the satellite underestimation is probably explained with the inhomogeneous atmosphere within the pixels which makes the NO_2_ VCD a combination of high (urban traffic station) and medium (urban background station) levels not always represented in the surface monitoring station network. Furthermore, the underestimation of the tropospheric satellite NO_2_ VCD in urban site could be related to the cloud properties included in the models which are used for the TROPOMI products retrieval as described in Compernolle et al. (2020) and Verhoelst et al. (2020). As supposed in Ialongo et al. (2020) to explain the overestimation of total TROPOMI VCD with respect to ground-based remotely sensed ones retrieved in low-level NO_2_ condition, the improvement of the NO_2_ products could be reached by replacing the surface reflectance climatology defined by OMI database (Kleipool et al. 2008). Indeed, the surface albedo information is derived from a monthly OMI climatology provided with a significantly lower spatial resolution than TROPOMI products and without the anisotropy of the surface reflectance described by the bi-reflectance directional function, now considered a high priority for coming improvement (van Geffen et al. 2019, p. 35). Besides, the rural background product could be affected by errors due to estimates of the stratospheric NO_2_ column and to uncertainties in the NO_2_ profile (van Geffen et al. 2019, p. 18). This effect is expected especially for the very low detected tropospheric NO_2_ VCD during the lockdown (low correlation coefficient, *R* = 0.20) while keeping the sensitivity to the temporal change of NO_2_ level occurring at the ground attested by the NO_2_ reduction retrieved from space, − 17%, and at the ground, − 20%.

## Conclusions

In the present work, atmospheric NO_2_ observations based on surface measurements and tropospheric TROPOMI VCD are shown and discussed in Rome and, for the first time, in the surroundings taking also the opportunity of the unprecedented NO_2_ decreases as a result of lockdown restrictions to contain the COVID-19. These restrictions have strongly reduced road traffic (− 67%), which is the main source of NO_2_ in the Rome area: other sources such as airplane and ship transportation had an even stronger reduction, while energy production was probably less affected by the restrictions.

Air pollution data from 20 monitoring stations of ARPA Lazio and from A. Liberti station of CNR-IIA have been used as input to assess the extent of changes on NO_2_, NO, O_3_, and CO levels across Rome and surroundings before (March–April 2019) and during the COVID-19 pandemic (March–April 2020). We further examined corresponding tropospheric TROPOMI NO_2_ VCD for the same periods and sites of surface measurements to evaluate the total effects of the minimum amount of emission sources due to reduced anthropogenic activities during the COVID-19 lockdown on air pollution. Monthly and diurnal variation analysis showed significant decreasing trends for NO_2_, NO, and CO concentrations over all monitoring stations in Rome and surroundings compared to previous year. The observed reductions in the compared periods were not highly determined by changes in meteorological conditions. The reduction in NO_2_ was higher in urban traffic (− 51%, range from − 30 to − 65%) than in urban background (− 34%, range from − 16 to − 52%) and rural sites (− 21%, range from − 10 to − 42%) due to restriction effects to contain the COVID-19 pandemic. A larger decrease occurred in NO levels, which reduced by − 56% (− 34 to − 76%) in urban traffic sites, − 48% (− 41 to − 61%) in urban background sites, and − 37% in rural background sites. Lower reduction was also observed for CO, while O_3_ levels recorded site-to-site variability showing both increases and decreases in urban and rural sites, respectively. Overall, the largest effect of the lockdown measures on concentrations of NO_2_, NO, and CO came from the large reduction in road transport and non-essential activities, as observed at urban traffic stations.

The TROPOMI products directly reveal the weakening of the NO_2_ hotspot in Rome and the NO_2_ reduction in the surrounding rural area during the lockdown. During 2019, the frequency distribution of the NO_2_ VCD for all the pixels including the monitoring stations in the rural background and in the city of Rome appears well-distributed and unimodal, becoming even more positively skewed distributed during the lockdown. Indeed, during the lockdown, the two distributions become light-tailed with an increase of pixel amount around the peaks especially in the urban site. Comparison between TROPOMI NO_2_ observations and surface measurements is performed taking advantage from the unprecedented high spatial resolution. The pixels including one or more monitoring stations are considered in order to explore the capability of this coupled system to monitor the spatial and temporal variations of NO_2_ in urban and rural sites. Firstly, the TROPOMI reductions obtained from the extracted urban pixels (− 43%) are consistent with the reduction provided by ESA (− 49%) for the entire city of Rome. Furthermore, the satellite reductions showed a sharp decline in NO_2_ larger in urban than in rural sites (− 17%) as retrieved with the concurrent surface measurements considering all the traffic and urban background stations (− 45%) and all the rural backgrounds (− 20%). A general underestimation of TROPOMI column density with respect to the surface concentration is retrieved in Rome for the inhomogeneous atmosphere not always accounted for by the monitoring stations and an overestimation in the low-level conditions in the rural environment is achieved.

In conclusion, the air pollution in Rome and surrounding areas was determined during the COVID-19 lockdown period combining satellite and surface observation.

The results are part of the groundwork to define a monitoring air pollution system composed of satellite atmospheric products and surface measurements with attention to the level of pollutant concentration. The unprecedented high spatial resolution of pollutants concentration from space leads to monitoring the air quality with more details in the urban area characterized by a mixing of traffic and backgrounds and also the influence of its emission sources in the surrounding rural environment where local contribution to the pollution is negligible.

Future efforts to combine the NO_2_ concentration measured at the surface and provided by TROPOMI with high spatial resolution could improve the air quality monitoring with a combined systems approach over polluted and rural environments.

Another interesting study will be the recovery of the air pollutants when the lockdown will be gradually released and the effect of new increase of different activities, such as industrial and transportation sectors on air quality, will be monitored by using satellite and surface observations.

Regarding air quality monitoring, this paper showed the need to resolve the uncertainties on quantifications and speciation of VOCs in Rome and surrounding areas. This is important not just for evaluating O_3_ variations and possible links between local air pollution and greenhouse gas concentrations in urban areas but also for projecting future changes for greenhouse gas mitigation and pollution prevention and abatement.

## Supplementary information

ESM 1 (PDF 2615 kb)

## Data Availability

All authors confirm that the data supporting the findings of this study are available within the article and its supplementary material. These data are available from the corresponding author on request.
