# Supplementary material for: Nitrogen dioxide reductions from satellite and surface observations during COVID-19 mitigation in Rome (Italy)
Source: Environ Sci Pollut Res Int. 2021 Jan 12;28(18):22981–3004. doi: 10.1007/s11356-020-12141-9 (PMC7801795; doi:10.1007/s11356-020-12141-9)
Supplement: Supplementary file 1 — (PDF 2615 kb) [file 11356_2020_12141_MOESM1_ESM.pdf]

Supplementary material for

Nitrogen Dioxide reductions from satellite and surface observations during COVID-19 mitigation in Rome (Italy)

Cristiana Bassani*, Francesca Vichi, Giulio Esposito, Mauro Montagnoli, Marco Giusto, and Antonietta Ianniello

CNR - Institute of Atmospheric Pollution Research, Via Salaria Km 29.3, CP10, 00015 Monterotondo S., Rome, Italy

*Corresponding author. Telephone: +39 0690672398; fax: +39 0690672660. E-mail address: cristiana.bassani@iia.cnr.it (C. Bassani).

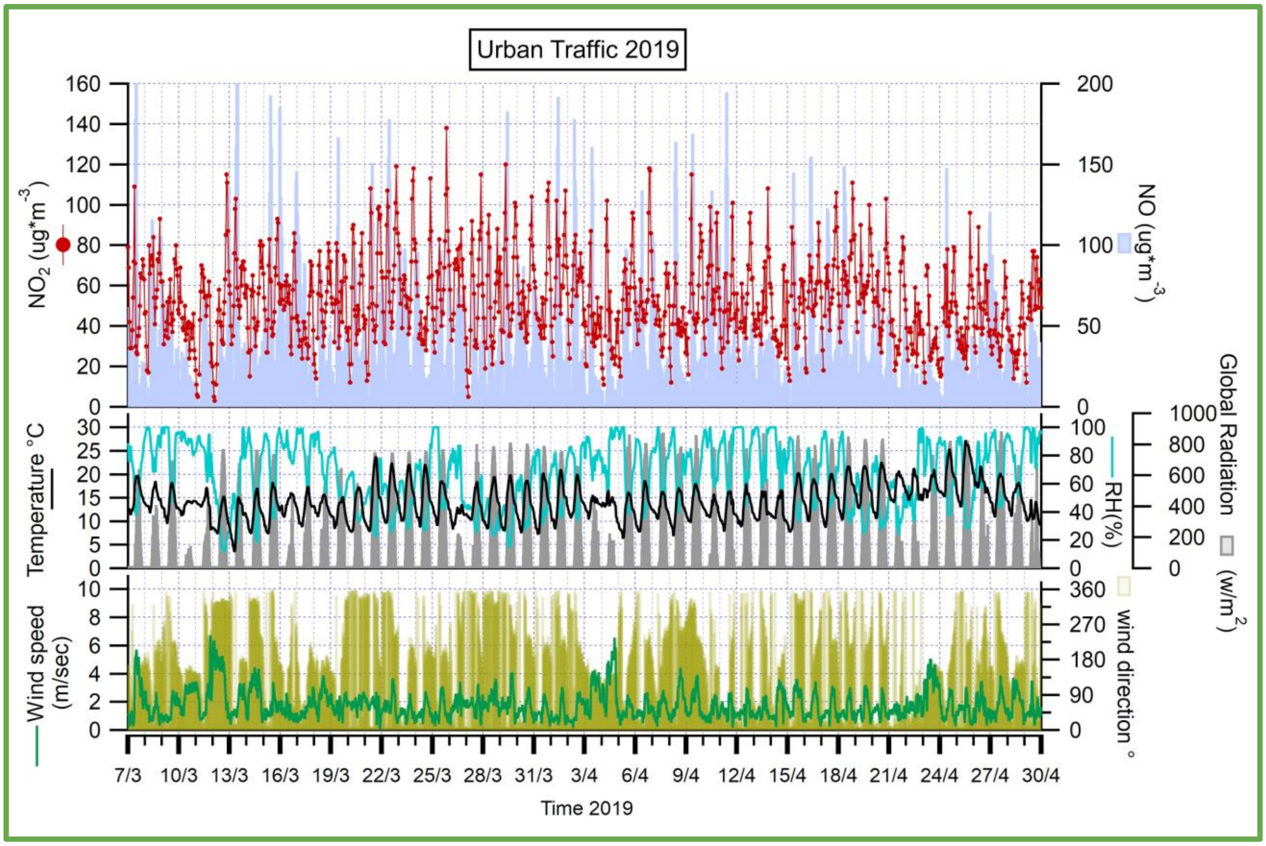

Fig. S1 Temporal trends of NO_2, NO, Temperature (T), relative humidity (RH), Global Radiation, Wind speed and direction in urban traffic monitoring station (Boncompagni) during March and April 2019

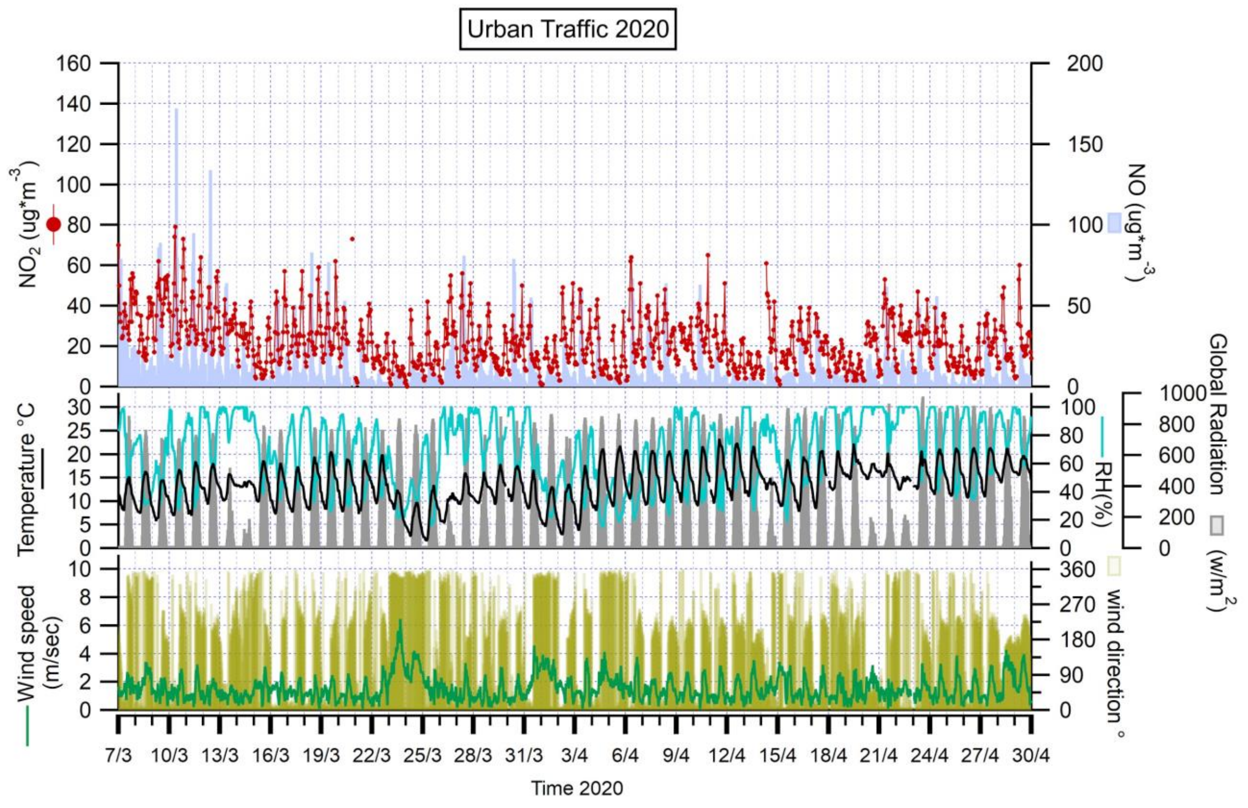

Fig. S2 Temporal trends of NO_2, NO, Temperature (T), relative humidity (RH), Global Radiation, Wind speed and direction in urban traffic monitoring station (Boncompagni) during March and April 2020

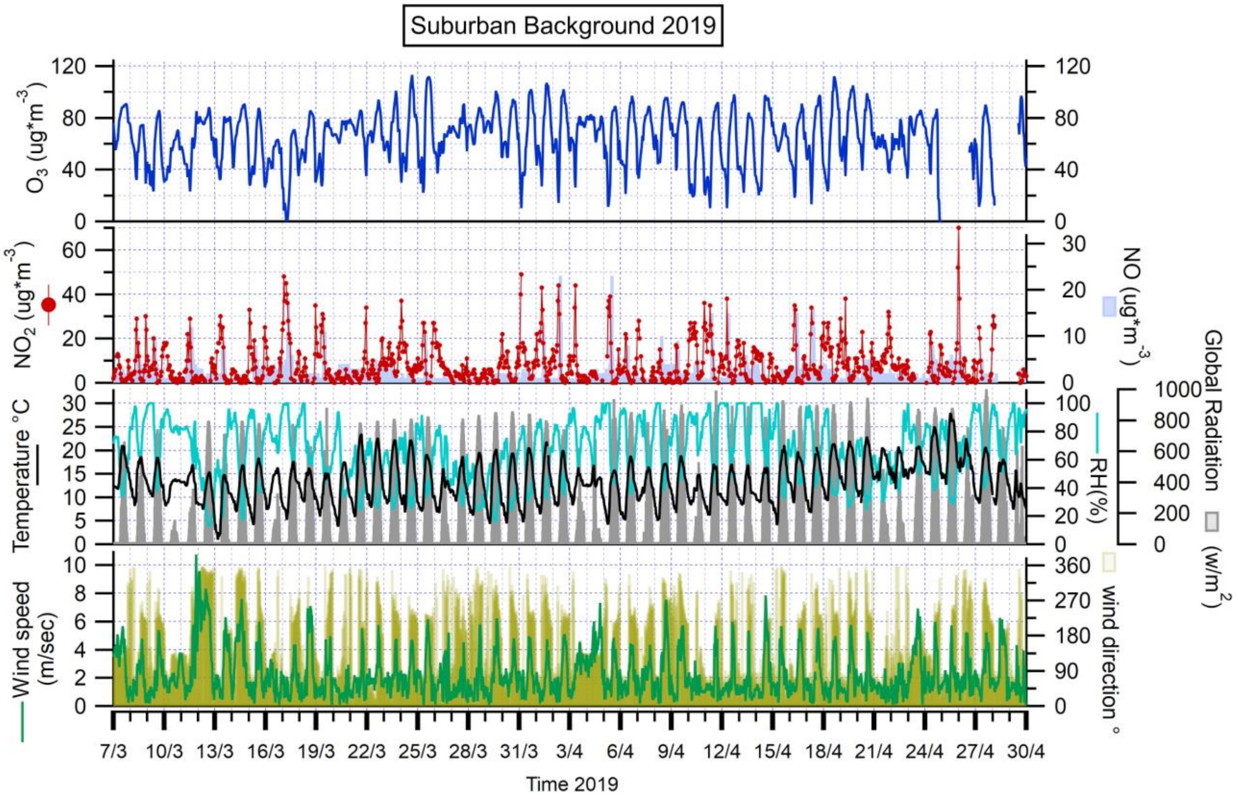

Fig. S3 Temporal trends of NO_2, NO, O_3, Temperature (T), relative humidity (RH), Global Radiation, Wind speed and direction in suburban background monitoring station (Cavaliere) during March and April 2019

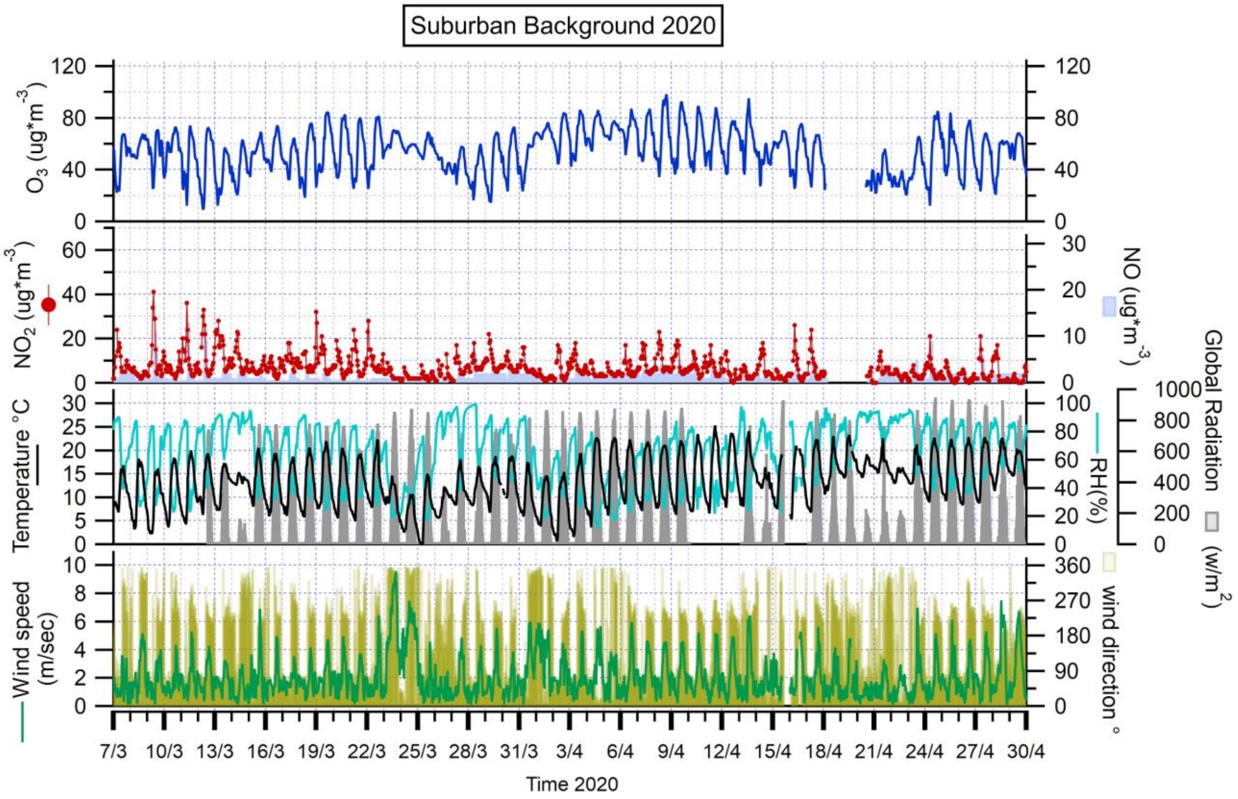

Fig. S4 Temporal trends of NO_2, NO, O_3, Temperature (T), relative humidity (RH), Global Radiation, Wind speed and direction in suburban background monitoring station (Cavaliere) during March and April 2020

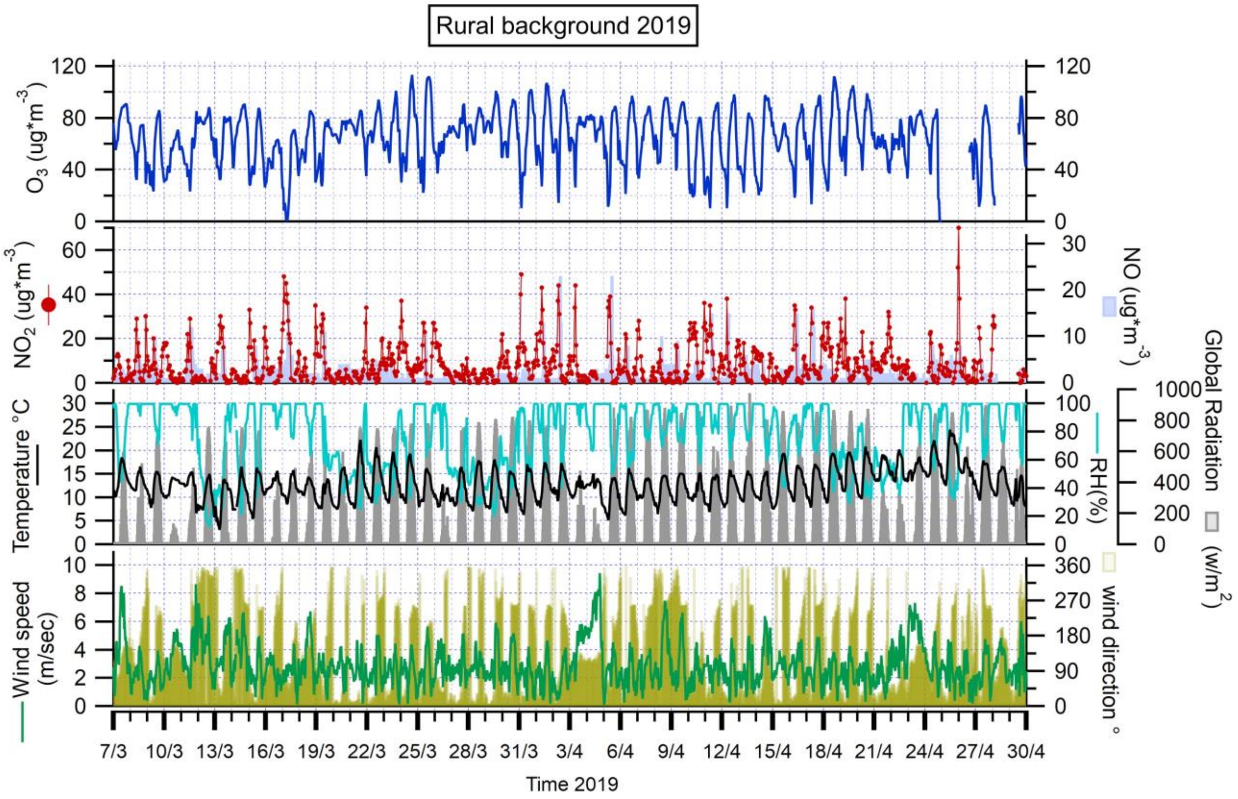

Fig. S5 Temporal trends of NO_2, NO, O_3, Temperature (T), relative humidity (RH), Global Radiation, Wind speed and direction in rural background monitoring station (Guido) during March and April 2019

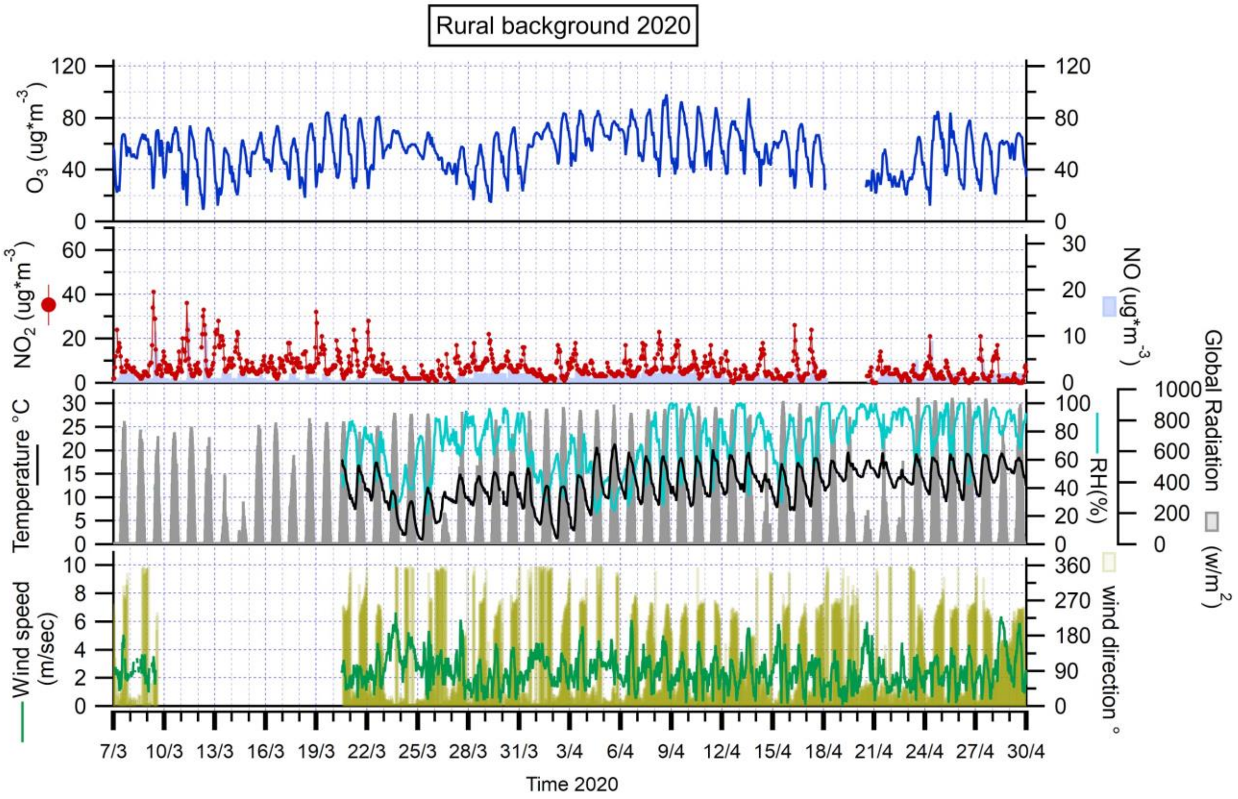

Fig. S6 Temporal trends of NO_2, NO, O_3, Temperature (T), relative humidity (RH), Global Radiation, Wind speed and direction in rural background monitoring station (Guido) during March and April 2020

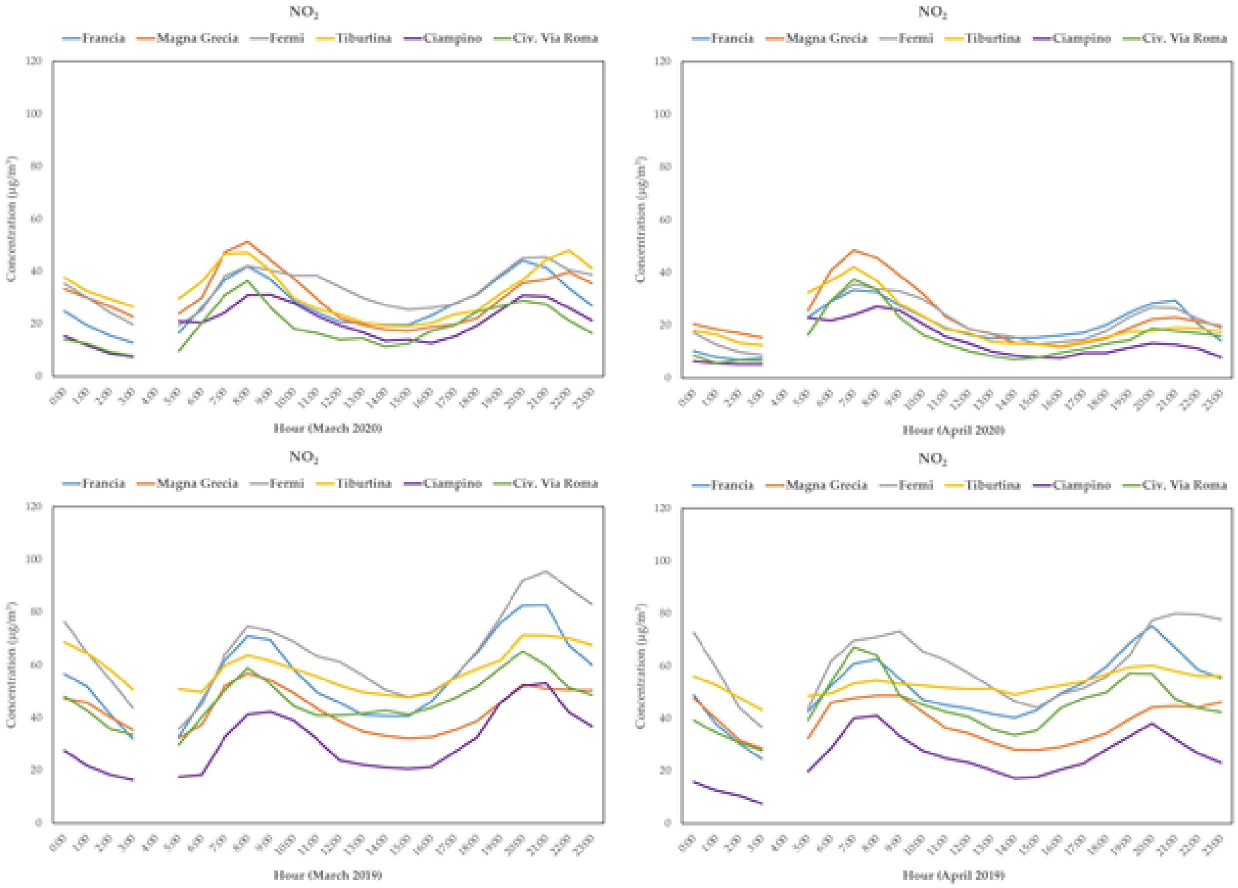

Fig. S7 Diurnal variations of the hourly mean NO_2 concentrations in six urban traffic monitoring stations during March 2020, April 2020, March 2019 and April 2019

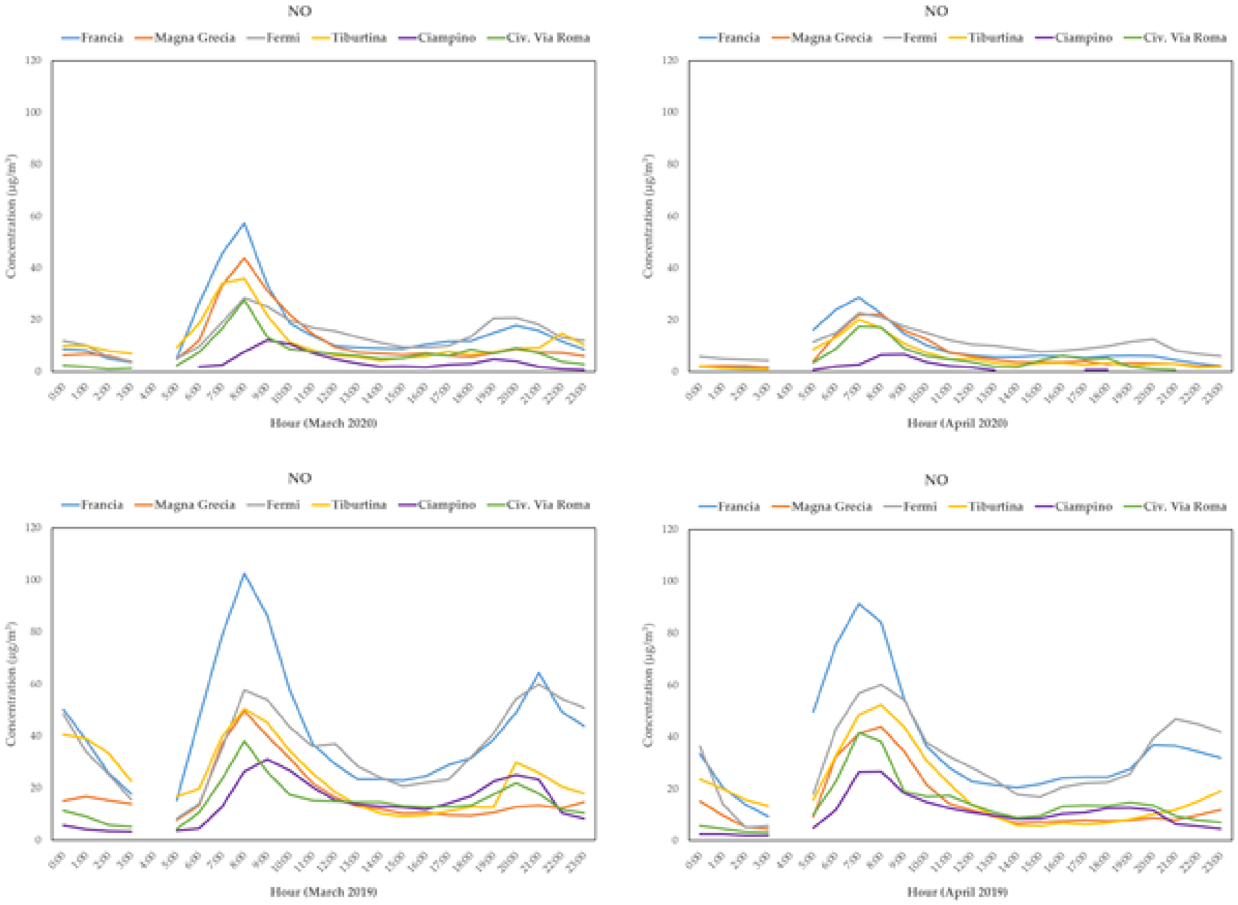

Fig. S8 Diurnal variations of the hourly mean NO concentrations in six urban traffic monitoring stations during March 2020, April 2020, March 2019 and April 2019

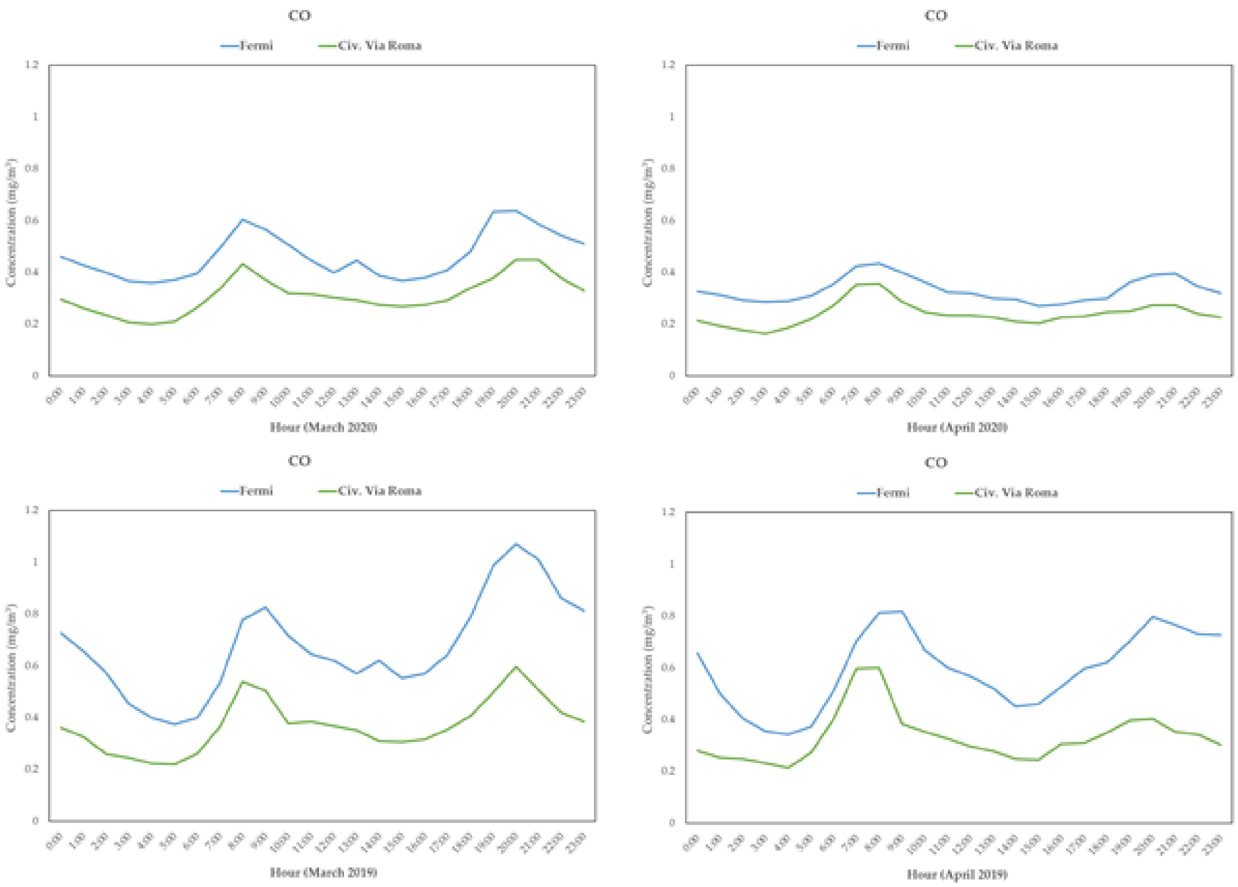

Fig. S9 Diurnal variations of the hourly mean CO concentrations in two urban traffic monitoring stations during March 2020, April 2020, March 2019 and April 2019

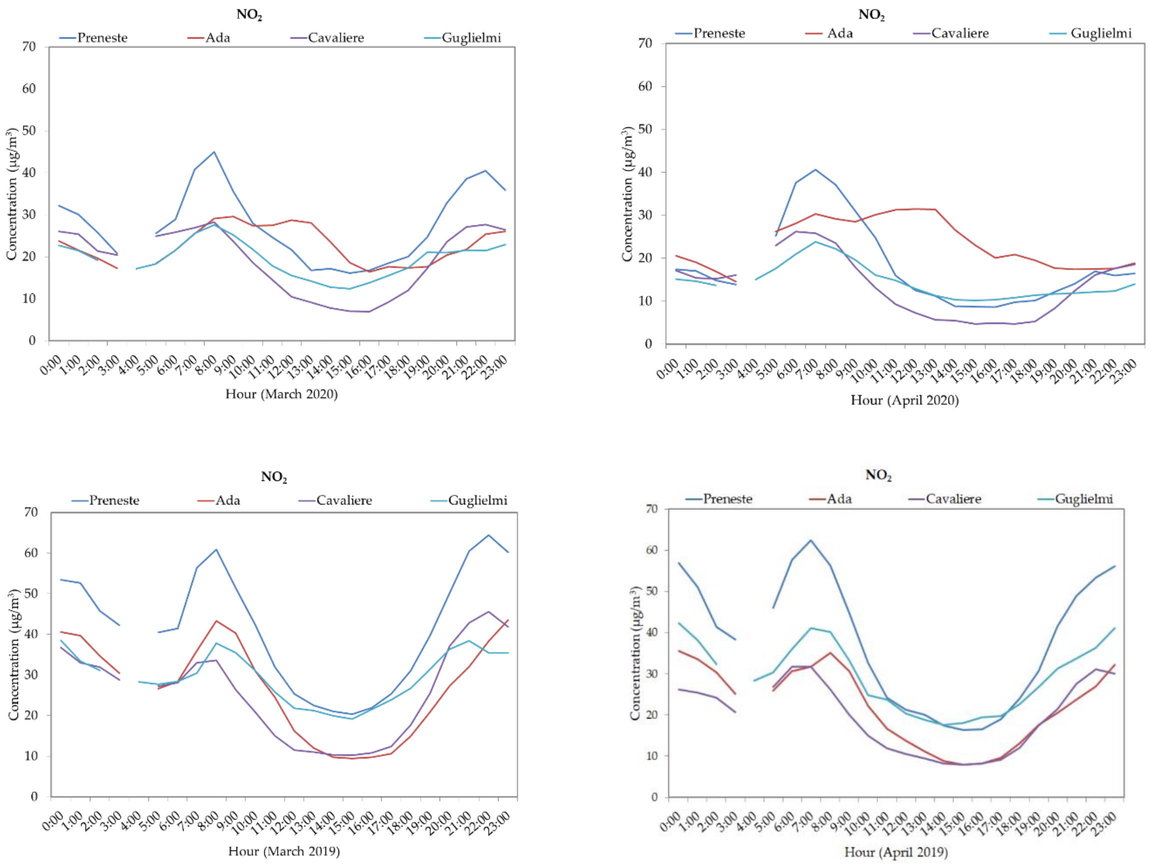

Fig. S10 Diurnal variations of the hourly mean NO_2 concentrations in the urban and suburban background monitoring stations during March 2020, April 2020, March 2019 and April 2019.

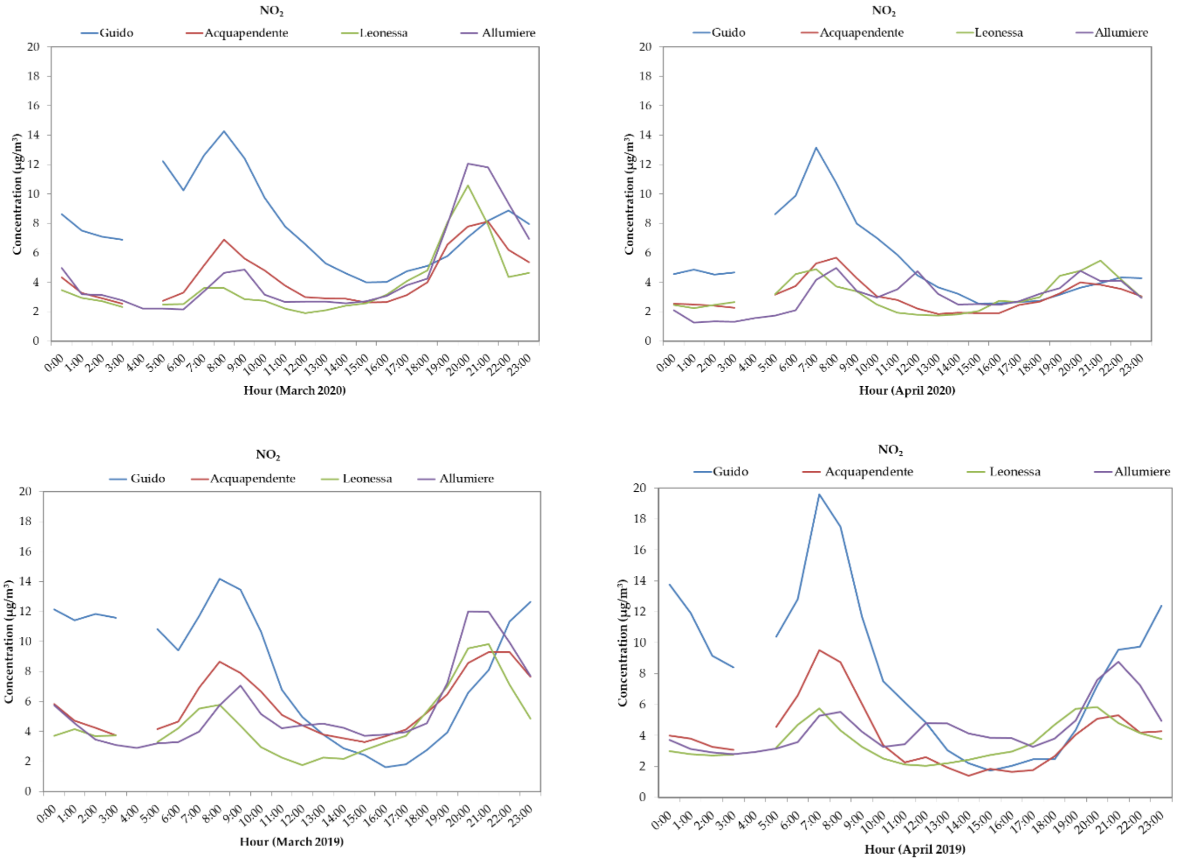

Fig. S11 Diurnal variations of the hourly mean NO_2 concentrations in rural background monitoring stations during March 2020, April 2020, March 2019 and April 2019.

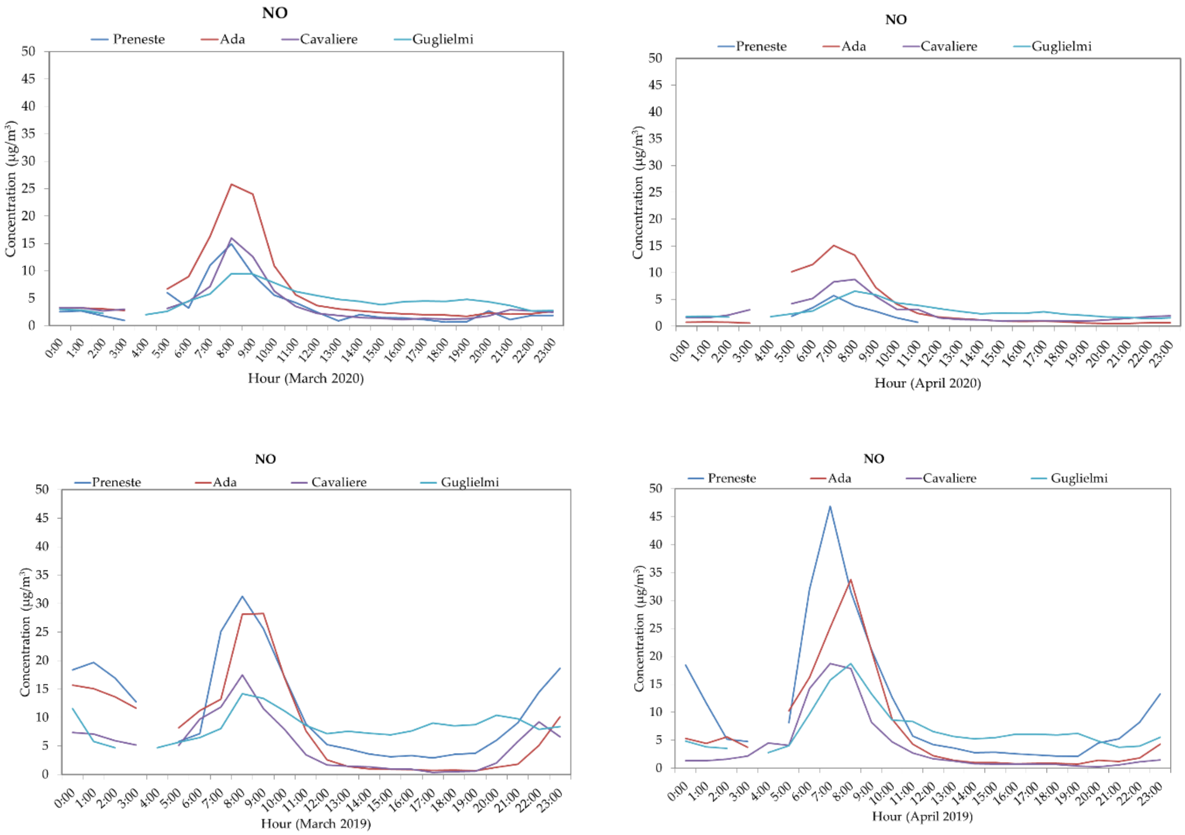

Fig. S12 Diurnal variations of the hourly mean NO concentrations in urban and suburban background monitoring stations during March 2020, April 2020, March 2019 and April 2019.

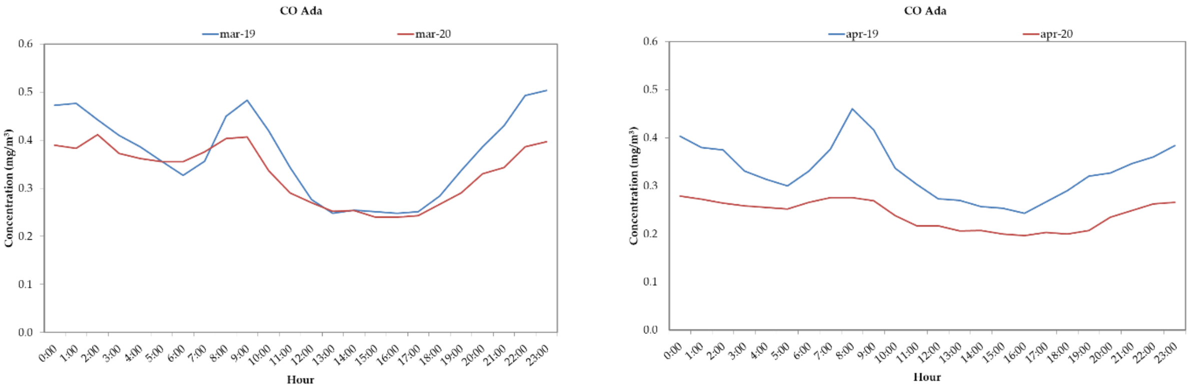

Fig. S13 Diurnal variations of the hourly mean CO concentrations in the urban background monitoring station of Ada during March 2020, March 2019 and April 2020, April 2019

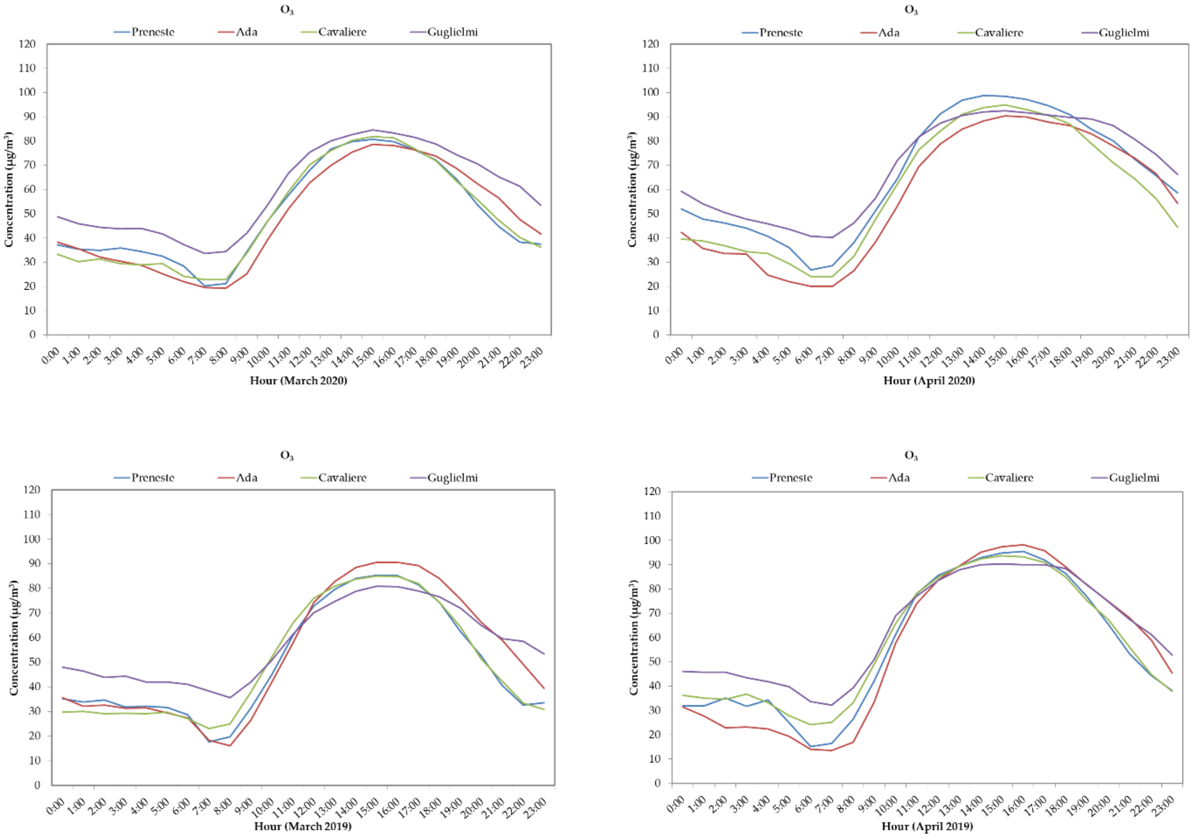

Fig. S14 Diurnal variations of the hourly mean O_3 concentrations in urban and suburban background monitoring stations during March 2020, April 2020, March 2019 and April 2019

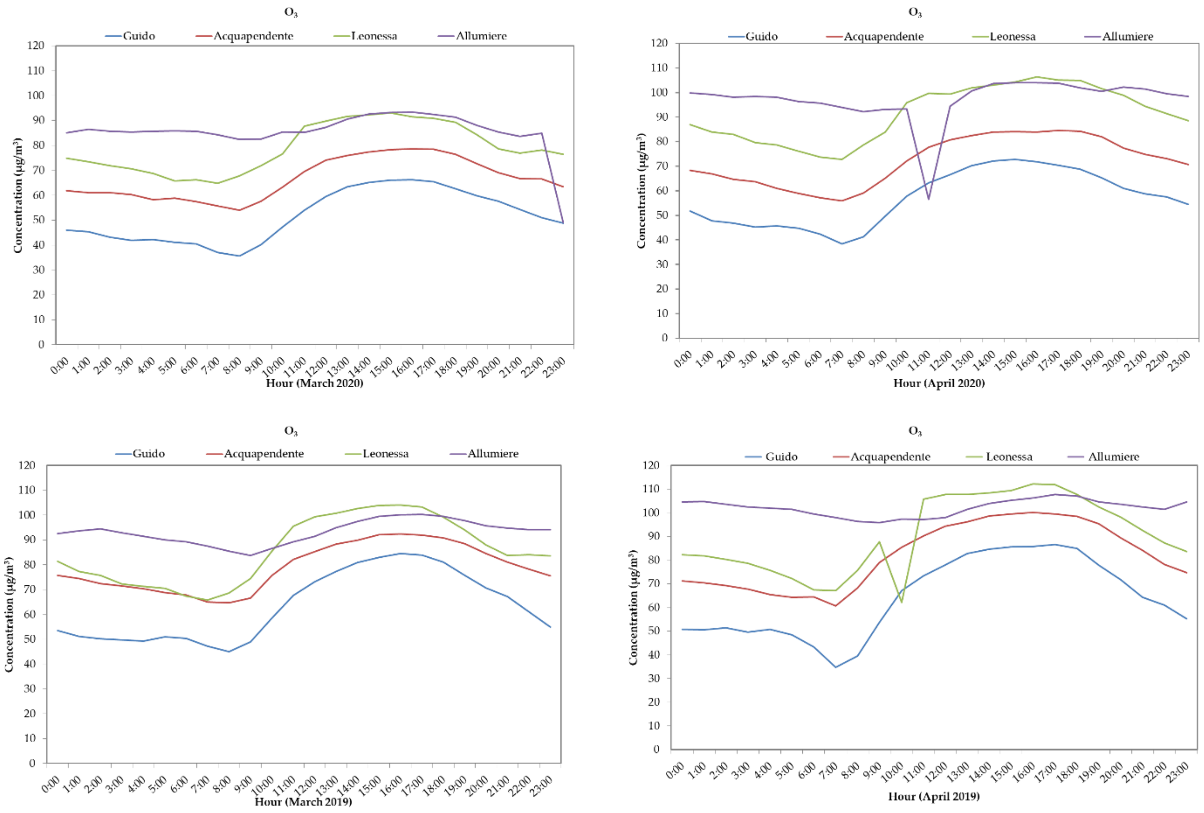

Fig. S15 Diurnal variations of the hourly mean O_3 concentrations in rural background monitoring stations during March 2020, April 2020, March 2019 and April 2019

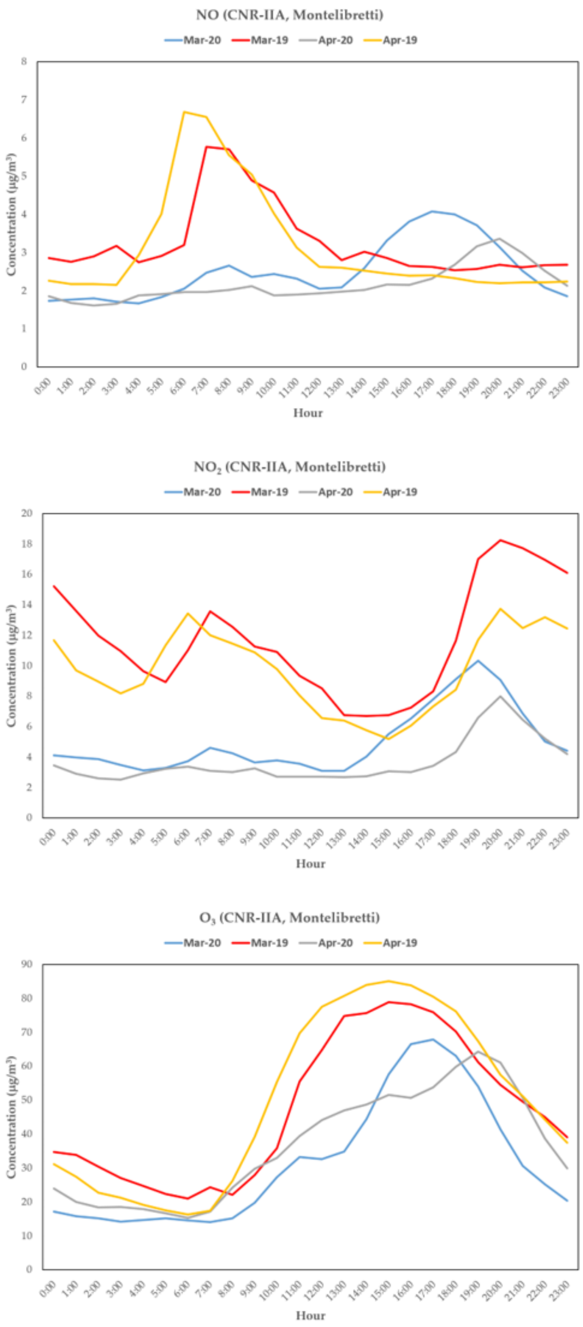

Fig. S16 Diurnal variations of the hourly mean NO, NO_2 and O_3 concentrations in A. Liberti station (CNR-IIA, Montelibretti) during March 2020, April 2020, March 2019 and April 2019

Table S1 Emissions of NO_2 (tons/year) in the targeted area (source: ARPA 2016)

**Emission macrosectors**	**Viterbo**	**Rieti**	**Rome**
Combustion in industry and energy plants	0.6	0.0	3716.3
Non-industrial combustion plants	572.5	472.7	4549.9
Production processes (combustion in the manufacturing industry)	740.8	65.3	2168.4
Production processes (contactless combustion)	139.7	1.5	382.1
Extraction and distribution of fossil fuels and geothermal energy	0.0	0.0	0.0
Use of solvents and other products	0.0	1.4	168.2
Road transport	1692.7	794.9	20167.4
Other moving sources and mobile machinery (off-road transport)	623.6	183.5	5104.2
Waste treatment and landfills	53.9	3.4	204.7
Agriculture	227.1	44.7	810.2
Other emissions and removals	5.0	17.5	169.9

Table S2 Average hourly car passes (source: Roma Mobilità 2020)

Location	March 2019	April 2019	March 2020	April 2020	Reduction compared to March 2019	Reduction compared to April 2019	Mean lockdown variation
Via Colombo - via del Canale della Lingua	262	229	103	35	-61%	-85%	-73%
Via Prenestina - via Togliatti dir. Centro	496	446	284	170	-43%	-62%	-52%
Via Togliatti - via Publicio	603	591	230	116	-62%	-80%	-71%
Via Tuscolana - via Arco di Travertino	1102	1054	511	129	-54%	-88%	-71%
Via Tuscolana - via Togliatti dir. Centro	1306	1283	573	289	-56%	-77%	-67%
Viale Marconi - p.le Edison	1564	1532	671	366	-57%	-76%	-67%
average	889	856	395	184	-56%	-79%	-67%

Table S3 Data on plane transportation of Fiumicino airport

Parameter	March 2019	April 2019	March 2020	April 2020	Reduction compared to March 2019	Reduction compared to April 2019	Mean lockdown variation
Number of planes	23931	26326	8624	2409	-64%	-91%	-77%
Number of passengers	3316724	3709509	634823	69423	-81%	-98%	-89%
Goods (tons)	15410	15669	6665	1874	-57%	-88%	-72%

Table S4 Data on ship transportation of Civitavecchia port

**Parameter**	**Jan-Jun 2019**	**Jan-Jun 2020**	**variation**
Liquid Bulk Goods (tons)	269400	348043	29%
Solid bulk goods (tons)	1144904	840320	-27%
Miscellaneous goods in packages (tons)	3209607	2408960	-25%
Ship berths	1450	912	-37%
Containers	62652	53582	-14%
Ferry passengers	550802	206562	-62%
Cruise passengers	1048664	173156	-83%
Vehicles	394947	219704	-44%

Table S5 National electric energy production from thermal plants

	**March 2019**	**April 2019**	**March 2020**	**April 2020**	**Reduction compared to March 2019**	**Reduction compared to April 2019**	**Mean lockdown variation**
National electric energy production from thermal plants (in GWh)	15418	14326	12948	11255	-16%	-21%	-19%

Table S6 O_3 changes during lockdown period averaged over March-April 2020 compared to the same periods averaged over previous years at urban, suburban and rural background stations, and Montelibretti station

Monitoring Stations	Mean O_3 during previous years (µg/m^3)	Mean O_3 during lockdown 2020 (µg/m^3)	Changes (%)
**UB***	48.35	54.65	13.45
**SB***	52.20	54.53	4.46
**RB****	79.05	75.25	- 5.71
**Montelibretti*****	37.50	33.86	- 9.83

* Mean value averaged over 4 previous years (2016-2019) at urban and suburban background station
** Mean value averaged over 3 previous years (2017-2019) at rural background station
*** Mean value averaged over 2 previous years (2018-2019) at Montelibretti station (CNR-IIA)
